# Comparative Analysis of Hypothalamic Responses to Stress and Glutamine Supplementation in Diet-Induced Obese Mice: A Study of Sex Differences

**DOI:** 10.1007/s10753-025-02428-9

**Published:** 2026-01-10

**Authors:** Virginie Dreux, Candice Lefebvre, Charles-Edward Breemeersch, Adam Tiffay, Pierre Déchelotte, Alexis Goichon, Ludovic Langlois, Moïse Coëffier

**Affiliations:** 1 Univ Rouen Normandie, INSERM, Normandie Univ, ADEN UMR1073 “Nutrition, Inflammation and Microbiota-Gut-Brain Axis”, F-76000 Rouen, France; 2 Univ Rouen Normandie, Institute for Research and Innovation in Biomedicine (IRIB), Rouen, F-76000 France; 3 CHU Rouen, Department of Nutrition and CIC-CRB1404, Rouen, F-76000 France

**Keywords:** Obesity, High fat diet, Glutamine, Stress, Hypothalamus, Neuroinflammation, Glial cells

## Abstract

Hypothalamic inflammation plays a key pathophysiological mechanism linking chronic consumption of a high fat diet (HFD) to the development of obesity and associated metabolic complications. Pilot studies report that oral glutamine (Gln) supplementation might reduce waist circumference and improve metabolic and inflammatory status in obesity patients. Although Gln metabolism plays a key role in intercellular communication in the central nervous system, its potential beneficial effects remain unexplored in these contexts. Here, we aimed to evaluate how stress and glutamine supplementation can modulate the hypothalamic response to HFD in mice using a chronic-restraint stress (CRS) model, which mimics IBS symptoms. From week 12 to week 14, mice received or not Gln diluted in drinking water (2 g/kg/day) and were placed in restraint tubes (2 h/day) for the last four consecutive days of protocol. Male and female obese mice showed a difference in vulnerability to CRS-induced effects. Moreover, mice responded to Gln supplementation in a sex-dependent manner, especially in stress conditions. Hypothalamic pathways regulating energy homeostasis were more impacted in male mice, whereas factors involved in neuroinflammation were more affected in female mice. Gln supplementation led to an increase in *Mc4r* and *Bdnf* mRNA levels and GFAP expression in male mice, while upregulated *Iba1* and *Il6* mRNA levels as well as signs of microgliosis were observed in stressed females. In conclusion, mice with obesity showed sex-specific hypothalamic response to glutamine supplementation and stress. Further investigations should be done to decipher underlying mechanisms.

**Supplementary Information:**

The online version contains supplementary material available at 10.1007/s10753-025-02428-9.

## Introduction

Obesity is a major public health concern that continues to increase in prevalence worldwide. In 2014, more than half of the European population was overweight and obese [28] with a projection of nearly one in two adults in the United States having obesity by 2030 [48]. This multifactorial disease involves a complex interaction between genetic, metabolic, and environmental factors, particularly the diet of modern populations and developed countries [47]. Obesity is characterized by chronic low-grade inflammation in both peripheral and central tissues. Indeed, energy imbalance and excess body fat lead to metabolic dysfunctions and activation of inflammatory pathways in peripheral organs such as adipose tissue, liver, pancreas, skeletal muscle but also in the central nervous system [16, 26]. Numerous studies have demonstrated neuroinflammation in several brain regions, including the hypothalamus, a critical structure involved in feeding behavior and control of energy homeostasis [17]. In particular, consumption of a high fat diet (HFD) used in diet-induced obesity (DIO) models induces early hypothalamic inflammation, which is maintained in long-term and is characterized by increased levels of pro-inflammatory markers, as well as dysfunction in crosstalk between neurons, astrocytes and microglia [12, 23, 35, 46]. Therefore, literature data suggest that neuroinflammation mediated by a reactive gliosis may be a central mechanism in obesity pathogenesis [37, 45]. Recently, we reported a sex-specific response to HFD at both peripheral [24] and central [13] levels. In particular, female mice showed earlier changes of astrocyte and microglial cell characteristics that may contribute to the resistance of females to HFD [13].

Furthermore, recent evidence highlights the importance to considering microbiota-gut-brain axis in the pathophysiology of obesity [3]. In addition, HFD consumption in rodents leads to intestinal hyperpermeability which is associated with other metabolic disorders such as type 2 diabetes mellitus (T2DM) and intestinal functional disorders [4, 32]. All of these peripheral alterations are also sex-specific [24]. Targeting microbiota and intestinal barrier function with *Akkermansia muciniphila* has previously been associated with an improvement in metabolic disorders [14, 34]. In the same way, pharmacological approaches using anti-inflammatory drugs with compounds able to tackle different inflammatory targets (anti-TNF-α, IL-6, IL-1 therapies) have already been tested with encouraging results [42]. However, the existence of a complex dynamic interplay between neuroinflammation and obesity requires further strategies.

Glutamine (Gln), the most abundant amino acid in plasma, shows anti-inflammatory effects that have been described for instance in the white adipose tissue, particularly through its action in the hexoamine pathway [11]. Indeed, reduced Gln levels during obesity lead to an increase in nuclear O-GlcNacylation in adipocytes that induces the transcriptional activity of pro-inflammatory pathways [33]. In addition, oral Gln supplementation reduced weight gain and improves insulin sensitivity in rats exposed to HFD [1]. Gln supplementation also reduces waist circumference and circulating levels of lipopolysaccharide (LPS) in adults with obesity [1]. Finally, Gln plays a key role in maintaining the integrity of the intestinal barrier by stimulating the synthesis of tight junction proteins or through other mechanisms [2]. Under stress conditions, Gln is also able to improve gut barrier function and intestinal disorders in both rodent models [15, 22] and humans [50]. Indeed, we have previously demonstrated that Gln is able to reduce colonic hyperpermeability induced by either water-avoidance stress [15] or a chronic restraint stress [22]. Interestingly, early-life stress induces a decrease of blood glutamine levels, potentially reducing its availability in the brain [51]. In addition, chronic immobilization stress is associated with mild cognitive impairments and neuronal damage in the hippocampus that are prevented by glutamine supplementation [5].

Taken together, Gln appears to be an interesting candidate for the development of nutritional strategies targeting the low-grade inflammatory response both in the periphery and in the central nervous system during obesity. We previously reported the impact of Gln supplementation on gut microbiota, intestinal permeability and peripheral inflammatory response in mice [24], bioRxiv preprint version, 10.1101/2024.11.21.624683) but the effects on neuroinflammation remain unknown, as do the differences between sexes to Gln supplementation. Given the anti-inflammatory properties of Gln, the aim of the present study was to evaluate whether oral Gln supplementation can affect the central inflammatory response in diet-induced obese mice under normal or stress conditions.

## Materials and Methods

### Animals

Animal care and experimentation were carried out in accordance with ARRIVE guidelines and the EU Directive 2010/63/EU. The experiments were approved by the regional ethics committee and authorized by the French Ministry of Higher Education, Research and Innovation (authorization on APAFIS #29283–2021012114574889 v5). For this study, two cohorts of 6-weeks-old C57BL/6J male and female mice were purchased from Janvier Labs (Genest-St.-Isle, France). Animals were acclimatized for 1 week and socially housed in a controlled environment (20 ± 2 °C with a 12- h light/dark cycle) with free access to water and food (standard diet, SD, 3.34 kcal/g with 14% from fat, 27% from proteins, and 59% from carbohydrates, #1314 formula, Altromin, Lage, Germany).

### Gln Supplementation

After acclimatization, one cohort of 7 weeks-old male and female mice (*n* = 48/sex, experiment 1, Supplemental Fig. S1) was first randomized into two groups receiving for 14 weeks either a SD or HFD (5.24 kcal/g with 60% from fat, 20% from proteins, and 20% from carbohydrates, #D12492i, Research Diet, New Brunswick, NJ, US). Diet compositions were previously detailed [13]. From week 12 to week 14, half of these animals fed with SD or HFD received Gln supplementation (G5792, Merck, Germany). Gln was diluted in drinking water to provide 2 g/kg of body weight per day. The Gln solution was prepared and replaced every day. The choice of the dose of Gln is related to previous studies [15, 25]). Thus, for each sex, this first cohort included 4 experimental groups: SD without Gln supplementation (SD), HFD without Gln supplementation (HFD), SD with Gln supplementation (SD + Gln), and HFD with Gln supplementation (HFD + Gln) (*n* = 12/group).

### Gln Supplementation and Chronic-Restraint Stress (CRS)

After acclimatization, a second cohort of 7 weeks-old, male and female mice (*n* = 36/sex, experiment 2, Supplemental Fig. S1) was fed with HFD for 14 weeks. At week 12, mice were randomized into three groups: HFD without stress (HFD); HFD with stress sessions (HFD + S) and HFD with stress and Gln supplementation (HFD + S + Gln). From week 12 to week 14, HFD + S + Gln group received Gln diluted in drinking water (2 g/kg/day). The Gln solution was renewed every day. Mice from HFD + S and HFD + S + Gln groups were briefly anesthetized with isoflurane and placed in restraint cages (Bioseb^®^, Vitrolles, France) for two hours a day before returning to their home cage (*n* = 12/group/sex). For CRS, the sessions were repeated for the last four consecutive days of the experiment (at the same hour) before sacrificing the animals. Control mice were kept in their home cage during the procedure. We used an experimental “IBS-like” model corresponding to a CRS to induce a psychological stress and reproduce the intestinal symptoms found in IBS. This approach was previously validated compared to an acute restraint stress [22].

Body weight was monitored weekly as well as before and after the Gln supplementation and the restraint stress sessions. Body composition was measured by EchoMRI on week 12 and week 14 of the protocol (EchoMRI, Houston, TX, US).

### Euthanasia and Sampling

For the 1 st experiment, mice were anesthetized two hours after the beginning of light cycle by intraperitoneal injection of ketamine/xylazine solution (100 and 10 mg/kg, respectively). Blood was collected from the inferior vena cava in heparinized tubes and centrifugated (3000 *g*, 4 °C for 15 min). Then, the plasma collected was immediately stored at −80 °C until use. After blood samples, mice were decapitated, and brains were harvested on ice either for dissection or postfixation. Hypothalamic dissected samples were snap frozen in liquid nitrogen and stored at −80 °C and whole brains were placed in a 4% paraformaldehyde (PFA) buffered solution.

For the 2nd experiment, mice were briefly anesthetized with isoflurane and decapitated after stress sessions. Blood samples were collected right after in heparinized tubes, centrifugated (3000 *g*, 4 °C for 15 min), and plasma was frozen at −80 °C. Brains were harvested on ice either for dissection or postfixation, as described above.

### RNA Extraction and Real-Time Quantitative Polymerase Chain Reaction (RT-qPCR)

Hypothalamic total RNA was extracted from frozen samples as previously described [13] (*n* = 32/sex for experiment 1; *n* = 18/sex, for experiment 2). Briefly, after reverse transcription of 1 µg of total RNA into cDNA by using 200 units of SuperScript™ II Reverse Transcriptase (Life Technologies, Cergy-Pontoise, France), qPCR was performed by SYBR™ Green technology on a BioRad CFX96 real-time PCR system (BioRad Laboratories, Marnes la Coquette, France). We focused on encoding-genes neuropeptides involved in food intake (*Npy*, *Agrp*, *Pomc*, *Mc4r*,* Crh*,* Bdnf*), pro-inflammatory (*Il1β*, *Il6*, *Tnfα*, *Cd11b*, *Nos2*) and anti-inflammatory (*Fizz1*, *Arg1*) profile markers as well as the glial markers (*Iba1*, *P2ry12*, *Gfap*). The mRNA relative levels of genes of interest were normalized using *Gapdh* and *Actb* as endogenous reference genes by dividing the starting quantity of genes of interest by the mean of reference genes. The specific primers were previously detailed [13].

### Staining by Immunofluorescence

Brains (*n* = 16/sex for experiment 1; *n* = 18/sex for experiment 2) were post-fixed in phosphate-buffered saline (PBS) containing 4% PFA for 24 h at room temperature (RT) followed by a cryoprotection step with a 30% sucrose solution (in 0.1 M PBS) for at least 24 h. Serial brain coronal Sect. (20 μm-thick) located between − 1.22 to −2.54 mm from bregma based on brain atlas coordinates (The Mouse Brain in Stereotaxic Coordinates, 3rd Edition, Franklin and Paxinos, 2008) were performed using a CM 1950 cryostat (Leica Biosystems). Sections were mounted on Superfrost™ Plus Adhesion microscope slides (Epredia, Portsmouth, US) and stored at −20 °C. Immunofluorescence experiments were carried out as previously described [13]. Briefly, slices were incubated with the primary antibodies: rabbit IBA1 polyclonal immunoglobulin G (IgG, Cat. No #019–19741, 1:2000, FUJIFILM Wako Pure Chemical, Osaka, Japan) and rat GFAP monoclonal IgG (Cat. No #13–0300, 1:500, Invitrogen, Rockford, US). Adequate Alexa Fluor 488-conjugated anti-rat (Cat. No #A11006, 1:400, Invitrogen,) and 555-conjugated anti-rabbit secondary antibodies (Cat. No #A21428, 1:400; Life Technologies) were used for fluorescence microscopy. Sections were mounted between slide and coverslip with the Fluoroshield™ with DAPI (Sigma Aldrich, Saint Louis, MO, US) medium and then stored at 4 °C in the dark until microscopic observation. Controls without primary antibodies were used to check the absence of nonspecific coupling of secondary antibodies.

### Image Acquisitions from Confocal Microscopy and 3D IMARIS Analysis

Images of 1024 × 1024 pixels (pixel size 0.23 μm) were acquired using a TCS SP8 DM6000B-CFS confocal microscope (PRIMACEN platform, Rouen, France) with a Leica DFC 365 Fx camera through a x63 oil-immersion lens. Z-stacks (0.3 μm steps) were taken in the arcuate nucleus (ARC) of the hypothalamus based on the shape of the 3rd ventricle (2 images/animal). All images were taken with the same confocal settings (zoom 0.75, pinhole, laser intensity, sequential mode). Raw files were then converted and analysed using IMARIS software (version 10.0.1, Oxford Instruments). First, the software was used to manually isolate cells using the cut function and to reconstruct the microglial surface and to obtain the cell volume using appropriate custom settings. Cells were included in the analysis when their soma was located within the central part of the z-stack and not cut by either the x or y plane. All morphological parameters were obtained after manually traced the IBA1 and GFAP staining using the Filament tracer mode. The volume as well as the number of total branch points and terminal points were measured for each cell. Branch Depth was defined as the number of branch points, or bifurcations, in the most complex path from the beginning point to a terminal point. Full Branch Level represented the highest value of Branching Level for the entire modeled cell. Filament Length corresponded to the sum of the lengths of all filaments. Statistical data from individual cells (3–4 cells/hemisphere/animal) were exported into Excel files and further analysed on GraphPad Prism software. In addition, Sholl analysis was performed from the filament reconstruction mode. To this end, spheres with a radius of 5 μm were superimposed starting at the center of the soma, and the number of process intersections that each sphere encountered was measured by the software.

### Cell Counting

Images (1936 × 1460 pixels, pixel size 0.23 μm) were obtained using a APOTOME ZEISS Axioimager Z1 fluorescence microscope. Z-stacks (1 μm-spaced) were taken from brain sections of 4 mice/group/sex with a ZEISS Axiocam CCD monochrome 160 camera and ZEN software (ZEISS Microscopy) at x20 magnification in the ARC (2 images/animal, *n* = 4 mice/group/sex). Identical illumination and exposure settings were applied for all images recorded. GFAP and IBA1 staining analysis were done on Image J software (National Institutes of Health, Bethesda, Maryland, US). The number of GFAP + and IBA1 + cells was manually counted in each hemisphere from maximal intensity projections of z-stacks.

### Statistical Analysis

All statistical analyses were performed using GraphPad Prism 8.0.1 (GraphPad Software Inc., San Diego, CA, USA). The ROUT method was used to identify outliers with a Q coefficient equal to 1%. For experiment 1, data were first compared with 2-way ANOVA (HFD x Gln) followed by Sidak’s post hoc tests, in a distinct sex manner. For experiment 2, HFD, HFD + S and HFD + S + Gln groups were compared, in a distinct sex manner, using unpaired *t* tests or Mann Whitney tests, except for Fig. 7 which results were analyzed using one-way ANOVA or Kruskal-Wallis test followed by multiple comparison tests. For the microscopy data, values were compared with nested *t* tests considering each subcolumn is for a replicate (animal) and each row in that subcolumn for a technical replicate (image or microglia cell/astrocyte). Sholl parameter was analyzed using 2-way ANOVA (distance x group) followed by multiple comparison tests. All graphs are presented as mean ± standard error of the mean (SEM) with the following significance levels: *p < 0.05, **p < 0.01, ***p < 0.001, ****p < 0.0001. Details for each performed tests are reported in the Supplemental table S1.

## Results

### Sex-specific Effects of Gln Supplementation on Hypothalamic Response in Obese Mice

**Fig. 1:**
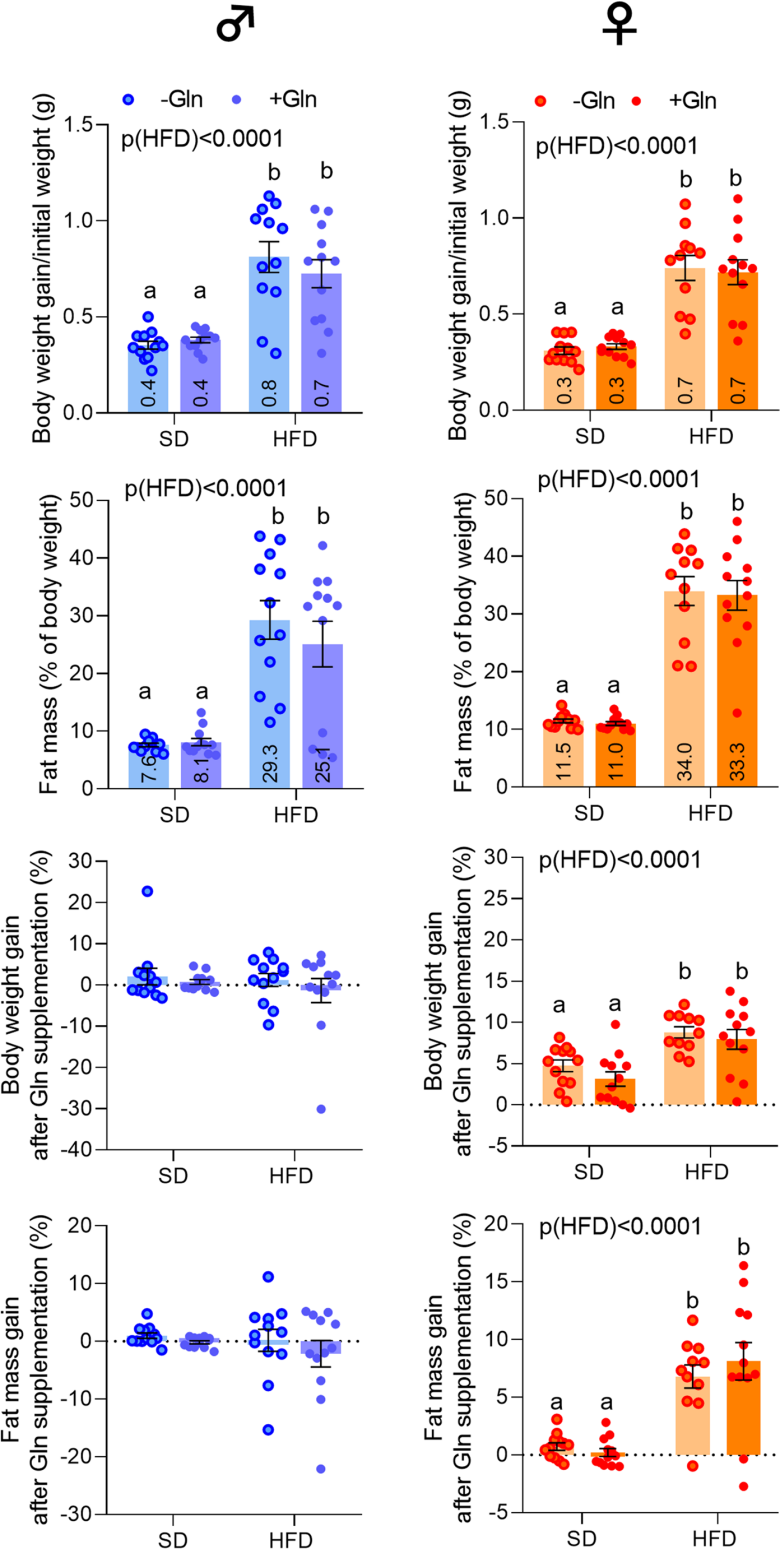
Effect of glutamine (Gln) supplementation on body weight and fat mass gains in mice. Ratio of body weight gain/initial weight (in g), fat mass level (in % of body weight) measured by EchoMRI at the week 14, body weight and fat mass gains (in %) after 2 weeks of Gln supplementation in drinking water (2 g/kg/day) from week 12 to week 14 in C57BL/6J male and female mice fed with either a standard diet (SD) or high fat diet (HFD) (experiment 1, *n* = 12/group). Data of these parameters have been taken and adapted from Lefebvre et al., [24] bioRxiv preprint version, 10.1101/2024.11.21.624683) and were compared with 2-way ANOVA (HFD x Gln) followed by Sidak’s multiple comparison tests : HFD vs. SD without Gln (-Gln) and with Gln (+ Gln), SD + Gln vs. SD and HFD + Gln vs. HFD. Values without a common letter (a, b) differ significantly (*p < 0.05). Data are presented as mean ± standard error of the mean (SEM) and plotted annotations in the bars correspond to the mean of each experimental group

**Fig. 2:**
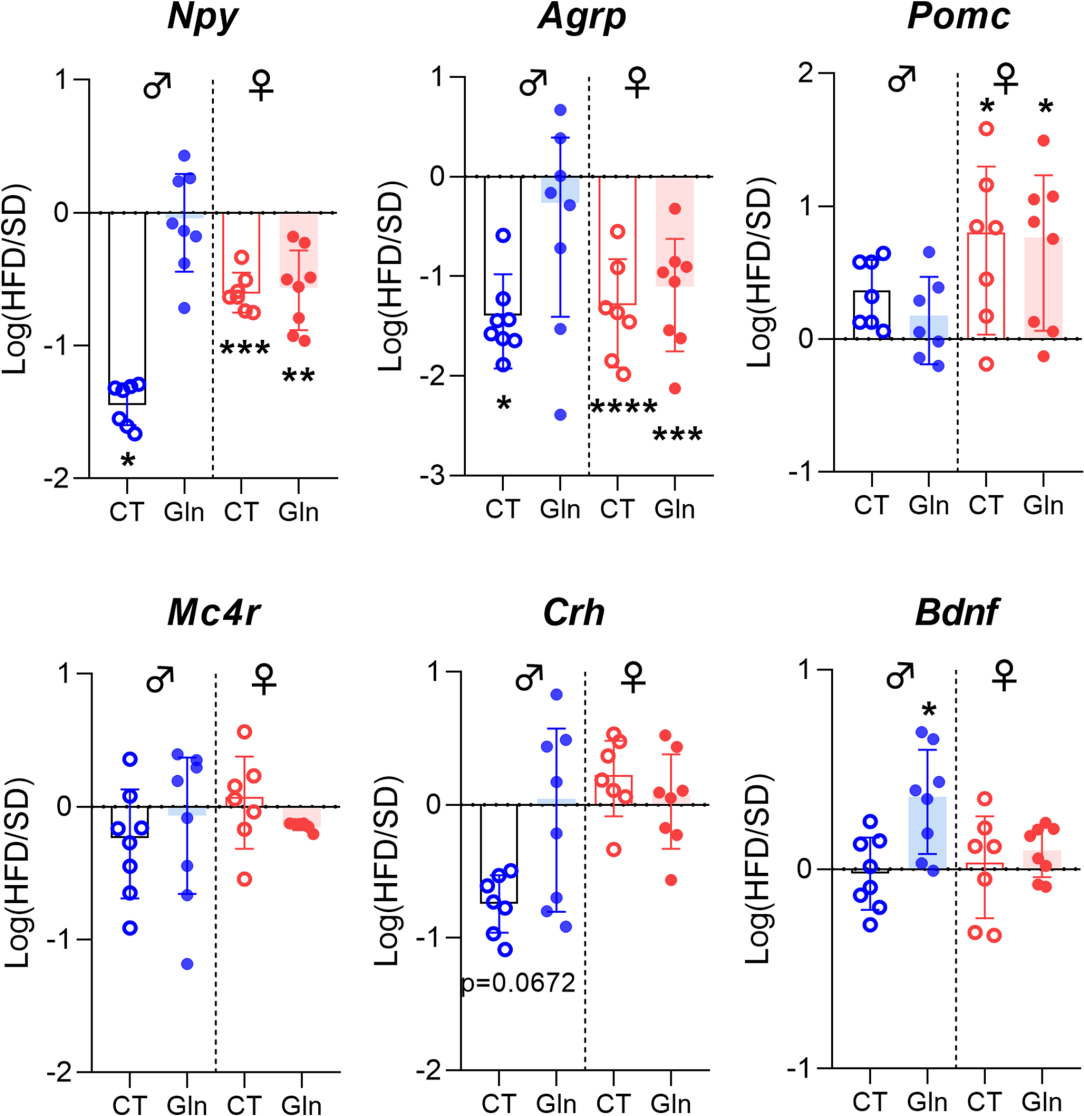
Effect of Gln supplementation on hypothalamic neuropeptides expression in mice. Relative quantification of mRNA transcript levels encoding orexigenic neuropeptides with neuropeptide Y (*Npy*) and agouti-related peptide (*Agrp*), anorexigenic neuropeptides with pro-opiomelanocortin (*Pomc*) and the melanocortin-4 receptor (*Mc4r*) as well as the corticotropin-releasing hormone (*Crh*) and the brain-derived neurotrophic factor (*Bdnf*) in hypothalamus of male and female mice fed with SD or HFD for 14 weeks (experiment 1, *n* = 8/group). All graphs show the fold changes (Log(HFD/SD)) of each HFD and HFD + Gln male and female mouse compared to the mean of their respective control group (SD and SD + Gln). Raw data were compared with 2-way ANOVA (HFD x Gln) followed by Sidak’s multiple comparison tests : HFD vs. SD without Gln (CT) and HFD with Gln vs. SD with Gln (*p < 0.05, **p < 0.01, ***p < 0.001, ****p < 0.0001). ***Npy*** : p(int) = 0.0665; p(HFD) = 0.0532 and p(HFD) < 0.0001 for male and female mice, respectively. ***Agrp*** : p(HFD) < 0.05 and p(HFD) < 0.0001 for male and female mice, respectively. ***Pomc*** : p(HFD) < 0.001 for female mice. ***Crh*** : *p* = 0.0792 HFD + Gln vs. HFD for male mice. ***Bdnf*** : p(int) < 0.05; p(HFD) < 0.0595; *p* < 0.05 HFD + Gln vs. HFD for male mice

As previously reported [13], male and female mice fed a HFD (M- and F-HFD, respectively) gained more weight (in g of initial body weight) and exhibited more fat mass (in % of body weight) after 14 weeks compared to control mice fed a SD (*p* < 0.0001, Fig. 1). After 2 weeks of Gln supplementation, HFD + Gln and SD + Gln groups had similar body weight gains/initial weight (in g) compared to their respective control groups (HFD and SD groups) in both male (0.73 and 0.38 vs. 0.81 and 0.35, respectively) and female mice (0.72 and 0.33 vs. 0.74 and 0.31, respectively, Fig. 1, Supplemental table S1). Similar results were observed for the fat mass, which did not show significant changes in Gln-supplemented animals in both male (25.10% and 8.11% vs. 29.28% and 7.63%) and female mice (33.27% and 11.02% vs. 33.97% and 11.48%) (Fig. 1, Supplemental table S1). Nevertheless, there were significant differences in the body weight and fat mass gain variations after Gln supplementation in female mice in contrast to male mice (Fig. 1). Next, we observed sex-specific effects of Gln supplementation on the hypothalamic response (Fig. 2). Indeed, in male mice, Gln supplementation blunted the HFD-induced significant decrease in mRNA levels of the orexigenic neuropeptides *Npy* and *Agrp* whereas mRNAs encoding the anorexigenic factors, *Pomc* and *Mc4r*, were not altered. Similarly, the reduction of *Crh* mRNA level induced by HFD was abolished by Gln supplementation. In contrast, Gln supplementation significantly increased *Bdnf* mRNA expression in HFD mice. All these Gln-induced changes were not observed in female mice (Fig. 2, p*-*values detailed in legends of figures, Supplemental table S1).

**Fig. 3:**
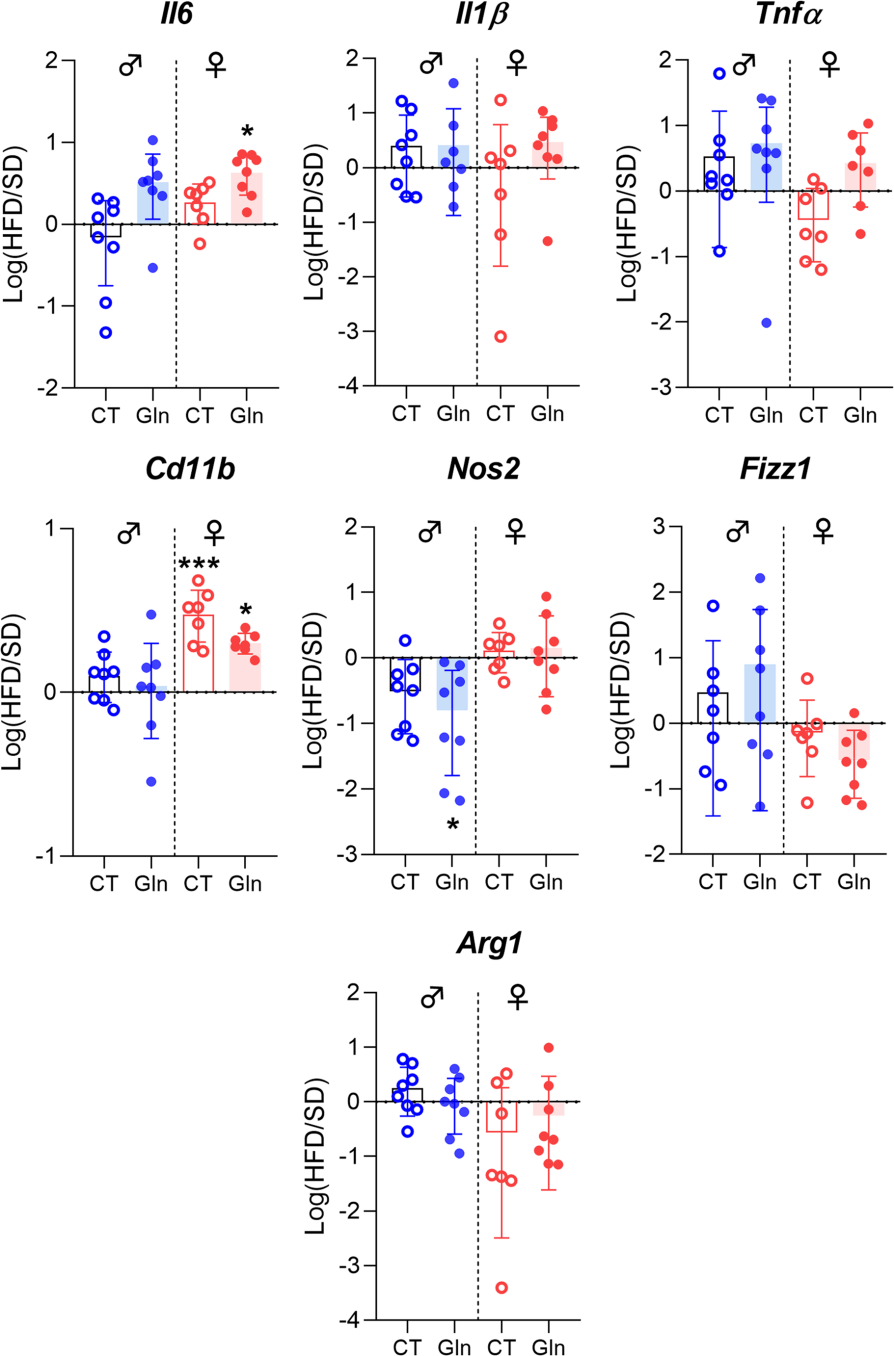
Effect of Gln supplementation on hypothalamic inflammatory markers expression in mice. Relative quantification of mRNA transcript levels encoding proinflammatory cytokines with interleukin-6 (*Il6*), interleukin-1β (*Il1β*), α- tumor necrosis factor (*Tnfα*), as well as M1 polarization markers (*Cd11b*, *Nos2*) and M2 polarization markers (*Fizz1*, *Arg1*) of macrophages in hypothalamus of male and female mice fed with SD or HFD for 14 weeks (experiment 1, *n* = 8/group). All graphs show the fold changes (Log(HFD/SD)) of each HFD and HFD + Gln male and female mouse compared to the mean of their respective control group (SD and SD + Gln). Raw data were compared with 2-way ANOVA (HFD x Gln) followed by Sidak’s multiple comparison tests : HFD vs. SD without Gln (CT) and HFD with Gln vs. SD with Gln (*p < 0.05, ***p < 0.001). ***Il6*** : p(HFD) < 0.01; p(Gln) < 0.05; *p* = 0.0652 SD + Gln vs. SD for female mice. ***Tnfα*** : p(HFD) < 0.05 and p(int) = 0.0752 for male and female mice, respectively. ***Cd11b*** : p(HFD) < 0.0001 for female mice. ***Nos2*** : p(HFD) < 0.01 for male mice. ***Fizz1*** : p(HFD) = 0.0698 and p(HFD) = 0.0981 for male and female mice, respectively

**Fig. 4:**
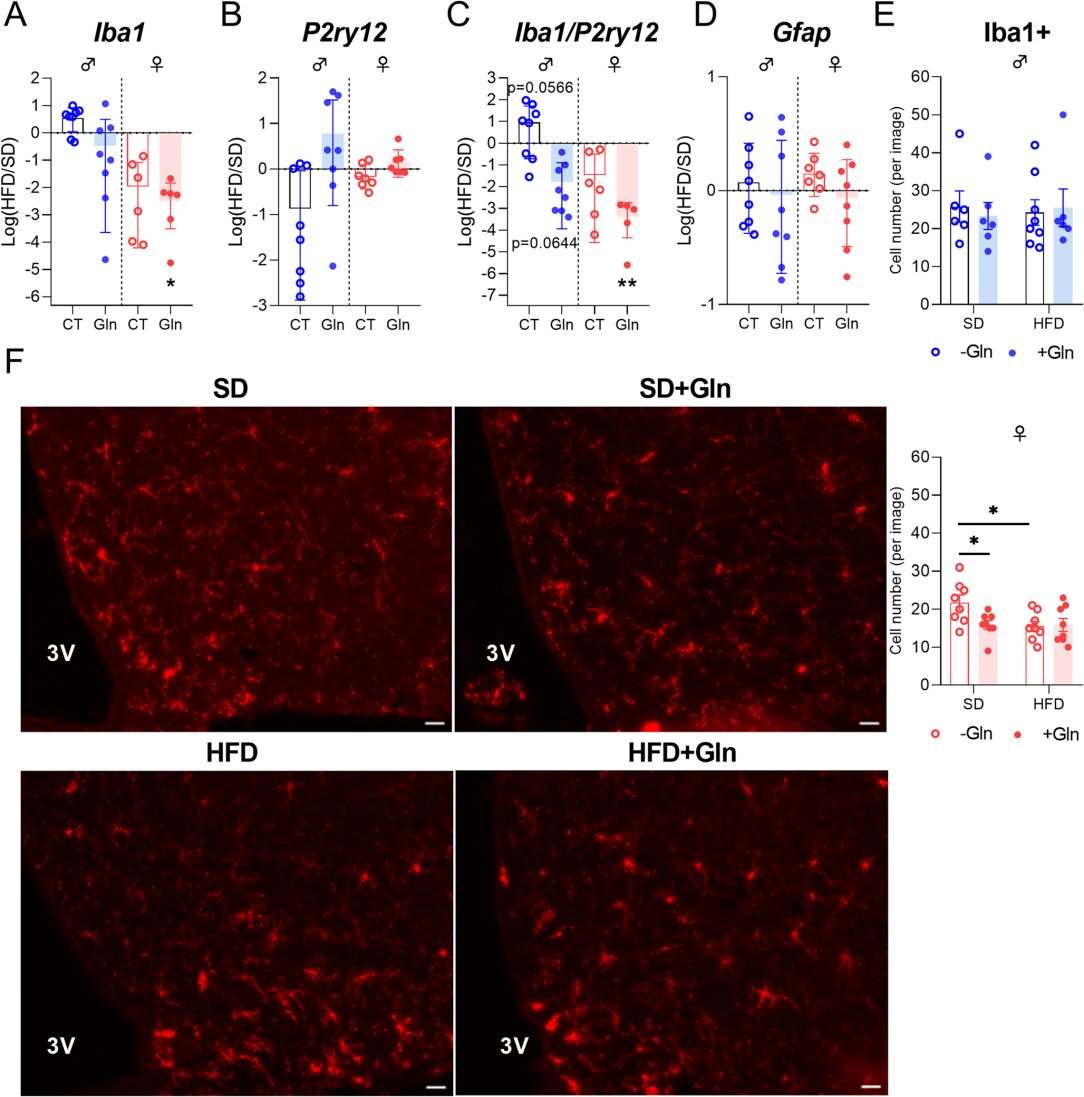
Effect of Gln supplementation on hypothalamic glial markers expression in mice. (**A**-**D**) Relative quantification of mRNA transcript levels encoding microglial markers with ionized calcium-binding adapter molecule 1 (*Iba1*), purinergic receptor P2Y12 (*P2ry12*) and astrocytic marker glial fibrillary acidic protein (*Gfap*) in hypothalamus of male and female mice fed with SD or HFD for 14 weeks (experiment 1, *n* = 8/group). All graphs show the fold changes (Log(HFD/SD)) of each HFD and HFD + Gln male and female mouse compared to the mean of their respective control group (SD or SD + Gln). Raw data were compared with 2-way ANOVA (HFD x Gln) followed by Sidak’s multiple comparison tests : HFD vs. SD without Gln (CT) and HFD with Gln vs. SD with Gln (**p < 0.01). ***Iba1*** : p(HFD) < 0.001; HFD vs. SD *p* = 0.0520 for female mice. ***P2ry12*** : p(int) < 0.05; *p* = 0.0741 HFD + Gln vs. HFD for male mice. ***Iba1/P2ry12*** : p(int) < 0.01; *p* < 0.01 HFD + Gln vs. HFD and p(HFD) < 0.01 for male and female mice, respectively. ***Gfap*** : p(Gln) < 0.05; *p* < 0.05 SD + Gln vs. SD for female mice. (**E**) Quantification of the number of immunopositive cells for Iba1 within the arcuate nucleus (ARC) from male and female mice fed with SD or HFD for 14 weeks which received or not Gln supplementation (experiment 1, *N* = 2 images/animal with *n* = 4 mice/group). Data were compared with nested *t* tests (*p < 0.05) and are presented as mean ± standard error of the mean (SEM). (**F**) Representative images of staining by immunofluorescence of Iba1 + cells manually and bilaterally counted within the ARC using Image J software in female mice (20 μm, −1.22 to 2.54 mm relative to Bregma). Scale bar : 20 μm, 3 V : third ventricle

**Fig. 5:**
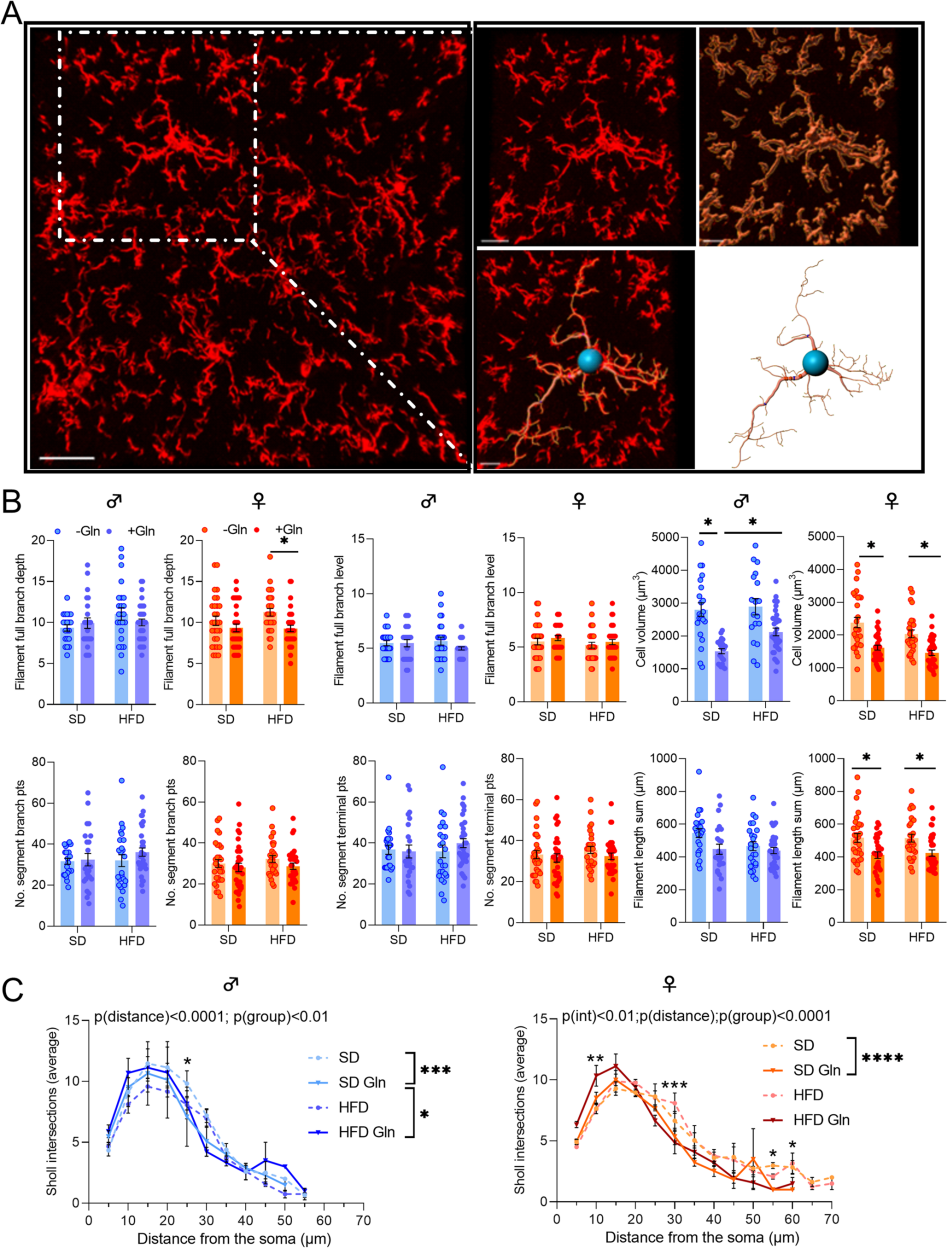
Effect of Gln supplementation on morphological parameters of microglial cells in mice. (**A**) First panel : representative image taken with confocal microscope showing Iba1 immunopositive cells obtained after maximal projection of Z-stacks within the ARC from female mice fed with HFD. Scale bar : 30 μm. Second panel : focus of the white square drawn in the first panel to highlight examples of surface reconstruction and filament tracing performed in IMARIS software on isolated microglial cell. Scale bar : 15 μm. (**B**) Graphs show the values of each cell individually analyzed for Filament full branch depth/level, Cell volume (µm^3^), Filament No. segment branch points/terminal points and Filament length sum (µm) in male and female mice fed with SD or HFD for 14 weeks which received or not Gln supplementation (*N* = 3–4 cells/hemisphere from *n* = 4 mice/group). Data were compared with nested *t* tests (*p < 0.05) and are presented as mean ± standard error of the mean (SEM). (**C**) Graphs show the mean distribution of the number of Sholl intersections as a function of the distance from the microglia soma for male and female mice at the week 14 (*N* = 3–4 cells/hemisphere from 4 mice/group). Values were compared with 2-way ANOVA (group x distance) followed by Tukey’s multiple comparison tests (*p < 0.05, **p < 0.01, ***p < 0.001, ****p < 0.0001). At 25 μm of distance from the soma, *p < 0.05 SD + Gln vs. SD for male mice. At 10 μm, **p < 0.01 HFD + Gln vs. HFD; at 30 μm, ***p < 0.001 HFD + Gln vs. HFD; at 55 μm, *p < 0.05 SD + Gln vs. SD; at 60 μm, *p < 0.05 SD + Gln vs. SD for female mice

**Fig. 6:**
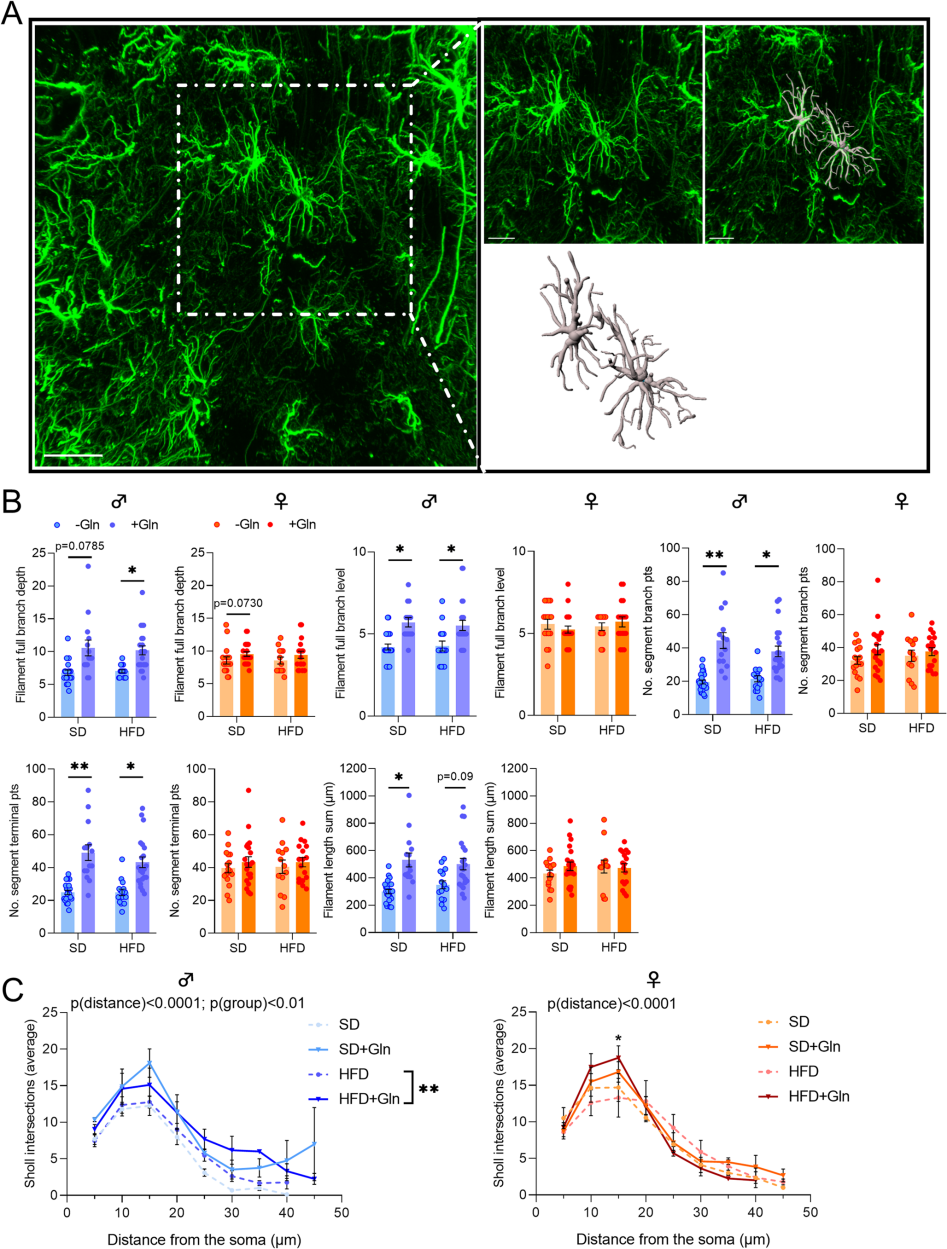
Effect of Gln supplementation on morphological parameters of astrocytes in mice. (**A**) First panel : representative image taken with confocal microscope showing GFAP immunopositive cells obtained after maximal projection Z-stacks within the ARC from male mice fed with HFD. Scale bar : 30 μm. Second panel : focus of the white square drawn in the first panel to highlight examples of surface tracing performed in IMARIS software on two isolated astrocytes. Scale bar : 15 μm. (**B**) Graphs show the values of each cell individually analyzed for Filament full branch depth/level, Filament No. segment branch points/terminal points, and Filament length sum (µm) in male and female mice fed with SD or HFD for 14 weeks which received or not Gln supplementation (*N* = 3–4 cells/hemisphere from *n* = 4 mice/group). Data were compared with nested *t* tests (*p < 0.05) and are presented as mean ± standard error of the mean (SEM). (**C**) Graphs show the mean distribution of the number of Sholl intersections as a function of the distance from the astrocyte soma for male and female mice at the week 14 (*N* = 3–4 cells/hemisphere from 4 mice/group). Values were compared with 2-way ANOVA (group x distance) followed by Tukey’s multiple comparison tests (*p < 0.05, **p < 0.01). At 15 μm, *p < 0.05 HFD + Gln vs. HFD for female mice

Regarding inflammatory response and glial reactivity, we first measured *Il6*, *Il1β* and *Tnfα* mRNA expressions. In both male and female mice, we did not observe significant effects of Gln on these mRNA levels, except for an upregulation of *Il6* in female HFD mice (Fig. 3, p*-*values detailed in legends, Supplemental table S1). Interestingly, *Iba1* mRNA expression and *Iba1/P2ry12* ratio were reduced by Gln only in female HFD mice whereas they were not significantly affected in female SD mice and in male SD mice (Fig. 4A, C, p*-*values detailed in legends). The number of Iba1-positive cells assessed by immunostaining in the in the arcuate nucleus was reduced in female mice by both Gln supplementation and HFD, without additive effects (HFD x Gln). No changes in the number of Iba1-positive cells were observed in male mice (Fig. 4E). *Cd11b* mRNA expression, another microglial marker, was upregulated by HFD in female mice but unaffected by Gln supplementation in both male and female mice, as were *Fizz1* and *Arg1* mRNA levels (Fig. 3, p*-*values detailed in legends, Supplemental table S1). In contrast, *Nos2* mRNA levels were reduced by Gln supplementation in male HFD mice but not in female mice (Fig. 3). The expression of the mRNA encoding the astrocyte marker, GFAP, was modified by Gln supplementation only in SD females (Fig. 4D, p*-*values detailed in legends, Supplemental table S1). Interestingly, the number of GFAP-positive cells was also decreased by Gln in female SD mice (Supplemental Fig. S2). To further characterize glial cells alterations, morphometric studies were performed using IMARIS software. Regarding microglial cells, cell volume was reduced by Gln supplementation in both male and female SD mice. This effect was also present in female Gln-supplemented HFD mice but was not significant in male Gln-supplemented HFD mice compared to HFD mice (Fig. 5B). In addition, Gln supplementation also reduced other parameters such as filament full branch depth and filament length sum only in female mice. In addition, we performed a Sholl analysis of individual 3D-reconstructed cells. Consistently, the microglial cells of Gln-supplemented mice displayed a significantly lower complexity level (indicated by a significant reduction of the average number of total Sholl intersections) (Fig. 5C). In contrast, astrocyte morphometric data were more markedly affected in male than in female mice (Fig. 6). Indeed, Gln supplementation increased all the parameters assessed in male mice in both SD and HFD conditions, whereas there was no significant difference in female mice (Fig. 6B).

In this first experiment, we showed sex-specific effects, albeit modest, of Gln supplementation on the hypothalamic response to Gln supplementation in SD- and HFD-fed mice. To go further, we performed a second experiment to assess the effects of Gln in response to a combination of HFD and CRS.

### Chronic Restraint Stress and Gln Supplementation Induced Sex-Specific Effects in the Hypothalamus of Obese Mice

**Fig. 7:**
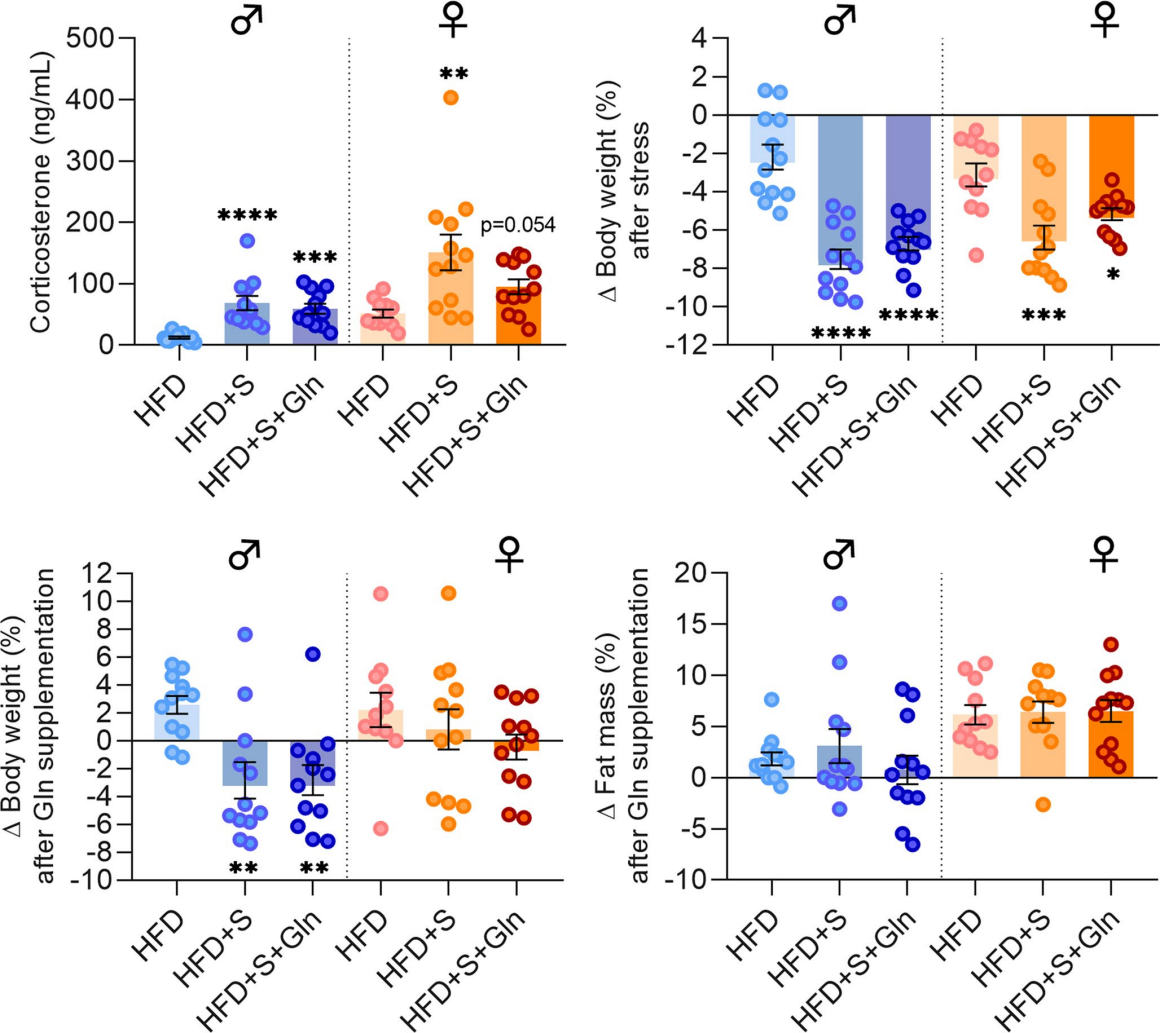
**Effect of stress and Gln supplementation on plasma corticosterone**,** body weight and fat mass in mice.** C57BL/6J male and female mice were fed with HFD for 14 weeks. From week 12, mice received or not Gln oral supplementation in drinking water (2 g/kg/day) for 2 weeks. The last 4 days of experiments, mice were subjected to a repeated restraint stress (S) for 2 h per day (experiment 2, *n* = 12/group). Corticosterone plasma levels (in ng/mL) as well as the body weight and fat mass variations (in %) were measured after stress and Gln supplementation at week 14. Data were compared with one-way ANOVA or Kruskal-Wallis tests followed by Tukey’s or Dunn’s multiple comparison tests (*p < 0.05, **p < 0.01, ***p < 0.001, ****p < 0.0001 vs. HFD) and are presented as mean ± standard error of the mean (SEM)

**Fig. 8:**
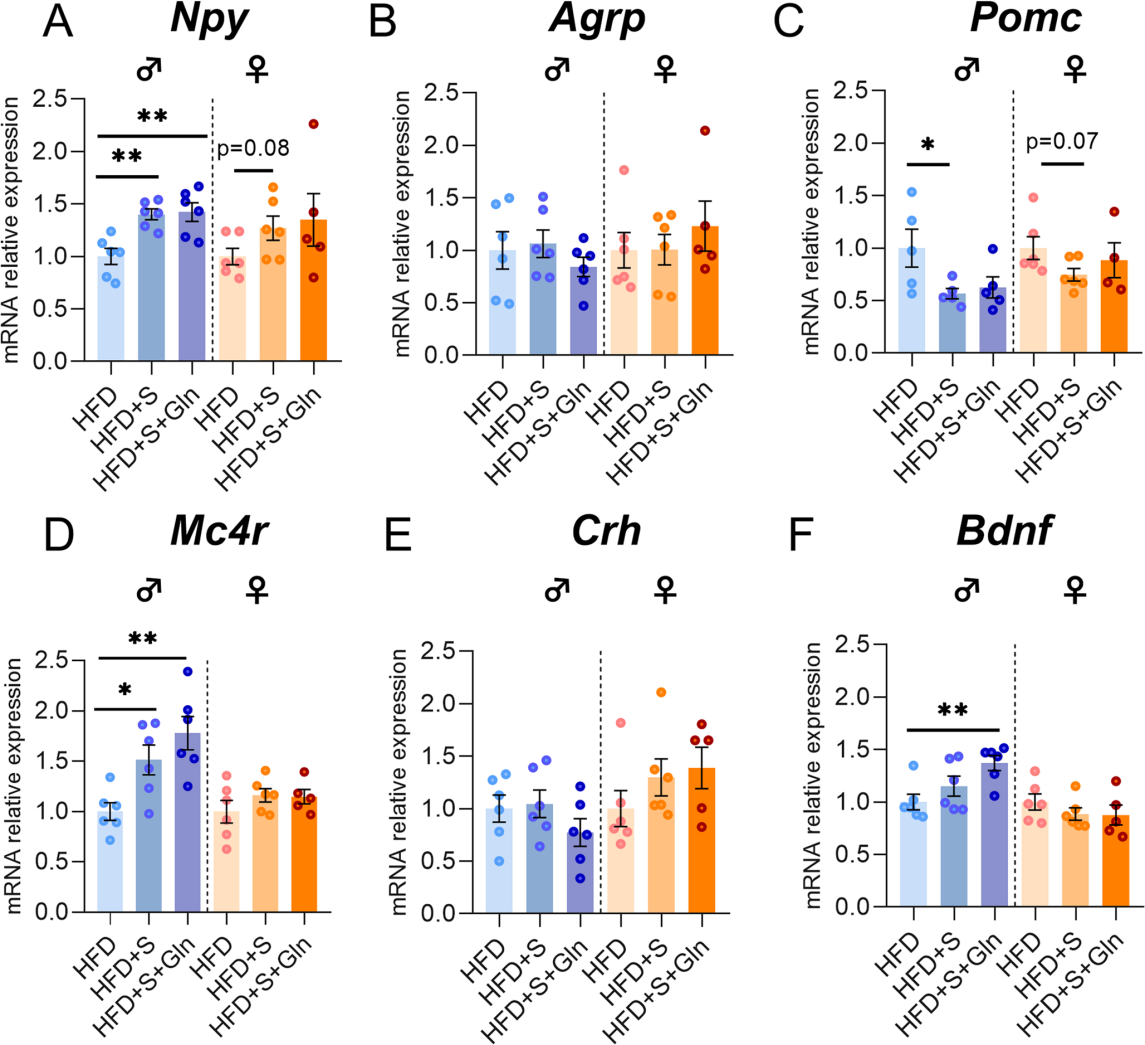
Effect of stress and Gln supplementation on hypothalamic neuropeptides expression in mice. (**A**-**D**) Graphs show the fold changes of mRNA transcript levels encoding orexigenic neuropeptides neuropeptide Y (*Npy*), agouti-related peptide (*Agrp*), anorexigenic neuropeptides pro opiomelanocortin (*Pomc*) and melanocortin-4 receptor (*Mc4r*) as well as the corticotropin-releasing hormone (*Crh*) and the brain-derived neurotrophic factor (*Bdnf*) (**E**, **F**) in hypothalamus of each HFD + S and HFD + S + Gln mouse compared to the mean of HFD group after 14 weeks (experiment 2, *n* = 6/group). All mRNA levels were quantified relative to *Gapdh* and *β-actin* housekeeping gene expressions. Data were compared with unpaired *t* tests or Mann Whitney tests (*p < 0.05, **p < 0.01) and are presented as mean ± standard error of the mean (SEM)

CRS was associated with a significant increase in plasma corticosterone concentrations in both male and female mice, which was partially attenuated only in female HFD + S + Gln. Interestingly, a similar pattern was observed for body weight loss (Fig. 7, p*-*values detailed in legends). Regarding the hypothalamic response and in particular mRNAs encoding factors involved in the regulation of food intake, only male HFD + S mice showed significant higher levels of mRNA encoding for *Npy* and *Mc4r* and significant lower levels of mRNA encoding for *Pomc* compared to HFD mice (Fig. 8A, C, D). Interestingly, no significant differences were observed in female HFD + S mice compared to HFD. *Agrp*,* Crh* and *Bdnf* mRNA levels remained unchanged in HFD + S mice (Fig. 8B, E, F). In both sexes, Gln supplementation did not affect these factors, except for a significant up-regulation in *Bdnf* expression in male mice (Fig. 8F, p*-*values detailed in legends).

**Fig. 9:**
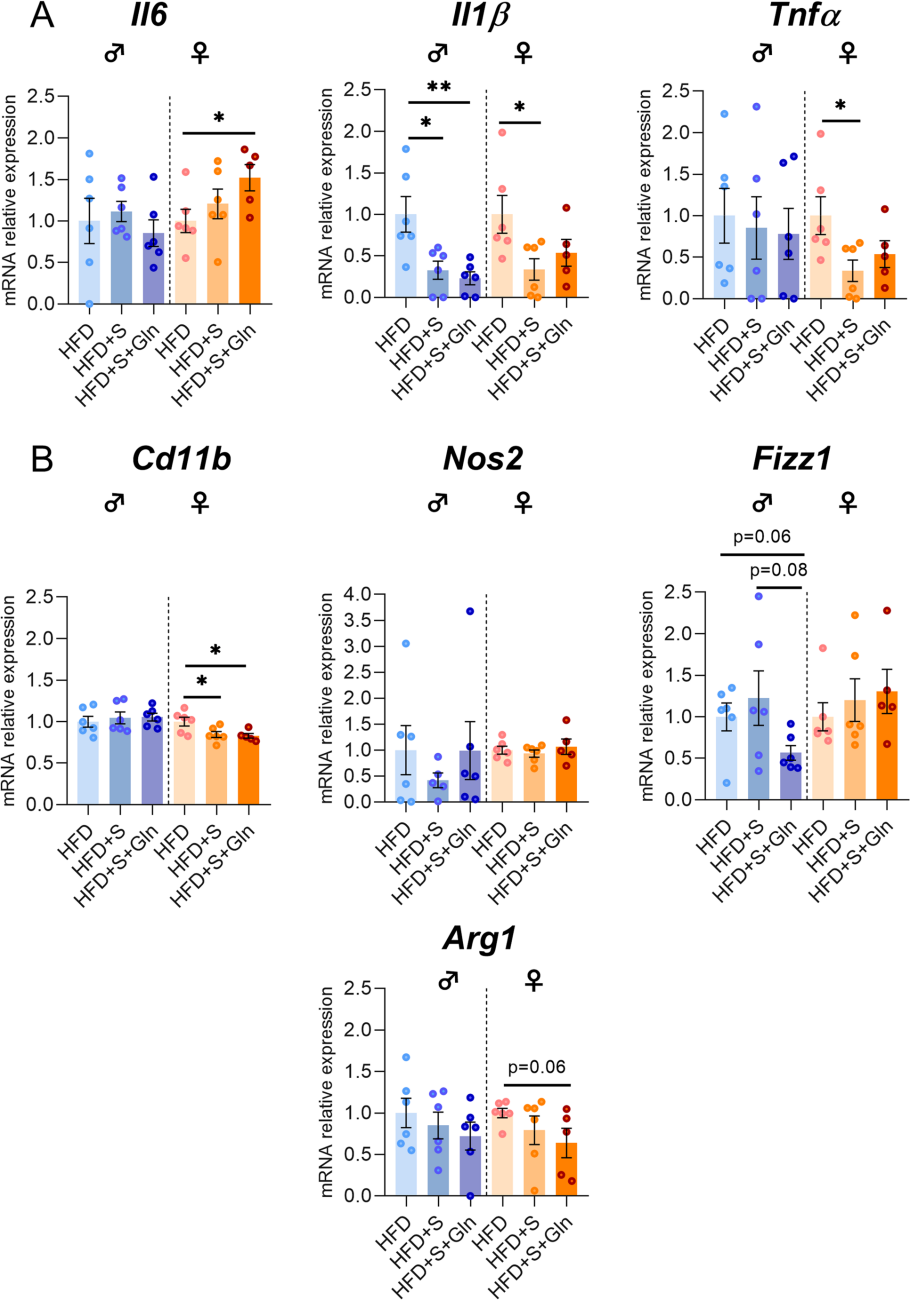
Effect of stress and Gln supplementation on hypothalamic inflammatory markers expression in mice. (**A**) Graphs show the fold changes of mRNA transcript levels encoding proinflammatory cytokines with interleukin-6 (*Il6*), interleukin-1β (*Il1β*), α- tumor necrosis factor (*Tnfα*) as well as M1 polarization markers (*Cd11b*, *Nos2*) and M2 polarization markers (*Fizz1*, *Arg1*) of macrophages (**B**) in hypothalamus of each HFD + S and HFD + S + Gln mouse compared to the mean of HFD group after 14 weeks (experiment 2, *n* = 6/group). All mRNA levels were quantified relative to *Gapdh* and *β-actin* housekeeping gene expressions. Data were compared with unpaired *t* tests or Mann Whitney tests (*p < 0.05, **p < 0.01) and are presented as mean ± standard error of the mean (SEM)

**Fig. 10:**
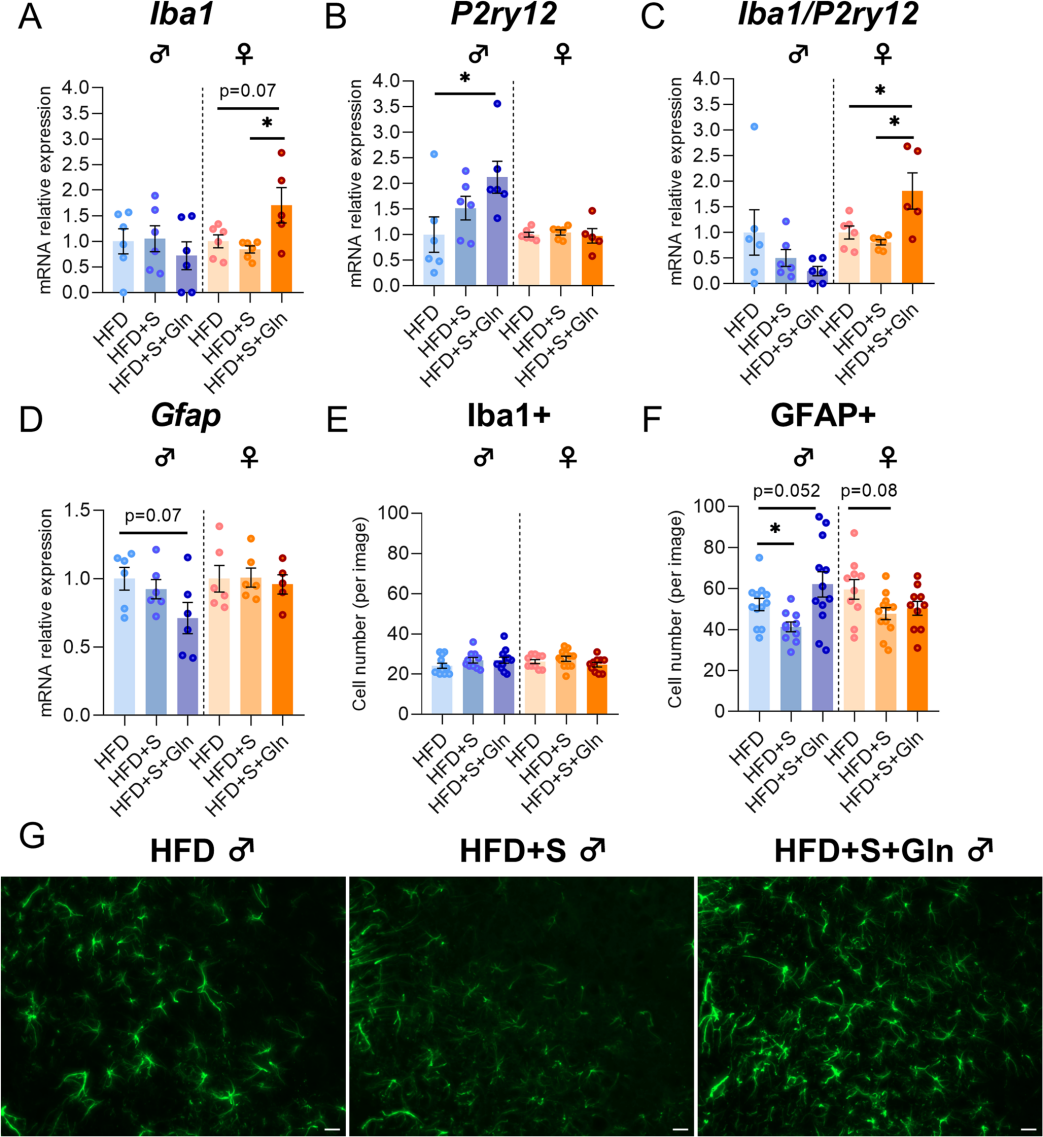
Effect of stress and Gln supplementation on hypothalamic glial markers expression in mice. (**A**-**D**) Graphs show the fold changes of mRNA transcript levels encoding microglial markers with ionized calcium-binding adapter molecule 1 (*Iba1*), purinergic receptor P2Y12 (*P2ry12*) and astrocytic marker glial fibrillary acidic protein (*Gfap*) in hypothalamus of each HFD + S and HFD + S + Gln mouse compared to the mean of HFD group after 14 weeks (experiment 2, *n* = 6/group). All mRNA levels were quantified relative to *Gapdh* and *β-actin* housekeeping gene expressions. Data were compared with unpaired *t* tests or Mann Whitney tests (*p < 0.05) and are presented as mean ± standard error of the mean (SEM). (**E**-**F**) Quantification of immunopositive cells for Iba1 and GFAP within the ARC from male and female mice fed with HFD for 14 weeks, subjected or not to the stress and which received or not Gln supplementation (experiment 2, *N* = 2 images/animal with *n* = 6/group). Values were compared with nested *t* tests (*p < 0.05) and are presented as mean ± standard error of the mean (SEM). (**G**) Representative images of staining by immunofluorescence of GFAP + cells manually and bilaterally counted within the ARC using imageJ software in male mice (20 μm, −1.22 to 2.54 mm relative to Bregma). Scale bar : 20 μm

**Fig. 11:**
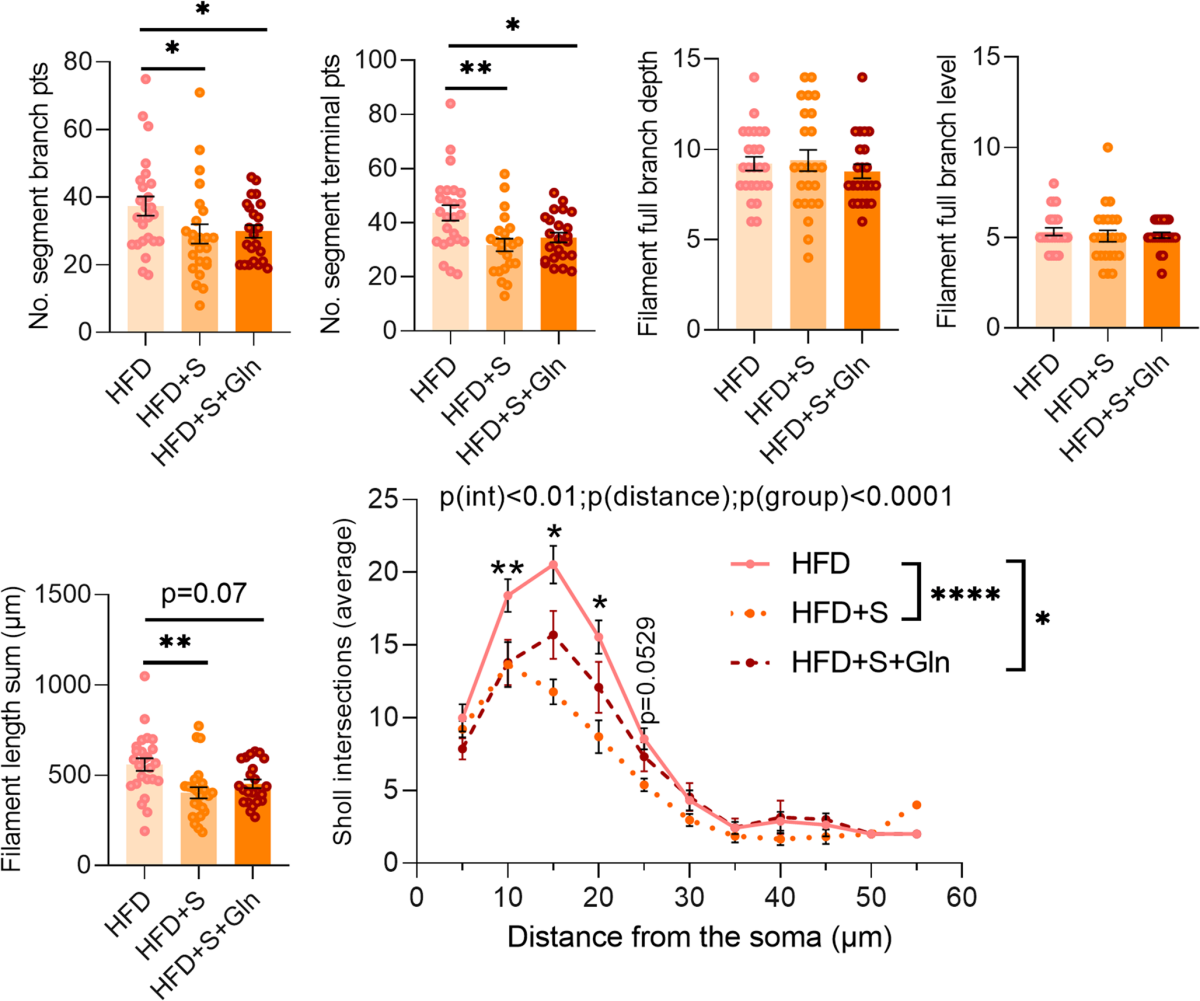
Effect of stress and Gln supplementation on the morphological parameters of astrocytes in female mice. Graphs show the values of each cell individually analyzed for Filament No. segment branch points/terminal points, Filament full branch depth/level, and Filament length sum (µm) in female mice fed with HFD for 14 weeks subjected or not to the stress and which received or not Gln supplementation (*N* = 3–4 cells/hemisphere from *n* = 6 mice/group). Data were compared with nested *t* tests (*p < 0.05, **p < 0.01) and are presented as mean ± standard error of the mean (SEM). Curves show the mean distribution of the number of Sholl intersections as a function of the distance from the astrocyte soma for female mice at week 14 (*N* = 3–4 cells/hemisphere 6 mice/group). Values were compared with 2-way ANOVA (group x distance) followed by Tukey’s multiple comparison tests (*p < 0.05, **p < 0.01, ****p < 0.0001). At 10 μm of distance from the soma, **p < 0.01 HFD + S + Gln and HFD-S vs. HFD; at 15 and 20 μm, *p < 0.05 between all groups; at 25 μm, *p* = 0.0529 HFD + S vs. HFD

Regarding the hypothalamic inflammatory and glial responses, we also observed sex-specific effects. Indeed, in male HFD + S mice, *Il1β* mRNA expression was significantly reduced compared to HFD mice (Fig. 9A) whereas other assessed factors including *Il6*, *Tnfα*, *Cd11b*, *Nos2*, *Arg1*, *Iba1*, *P2ry12* and *Gfap*, were not significantly affected. In contrast, not only *Il1β* mRNA expression, but also *Tnfα* and *Cd11b* mRNA levels in female HFD + S mice were significantly reduced (Figs. 9 and 10). Under these stress conditions, Gln supplementation increased *P2ry12* and reduced *Gfap* and *Fizz1* mRNA levels in male mice. In contrast, in female mice, Gln supplementation partially reversed *Il1β*, *Tnfα* and decreased *Arg1* and increased *Il6* mRNA expressions while *Cd11b* mRNA was not altered by Gln (Fig. 9). Interestingly, Gln supplementation also increased *Iba1* mRNA expression and consequently *Iba1/P2ry12* ratio in female HFD + S + Gln compared to HFD and HFD + S mice (Figs. 9 and 10). Under these conditions, the number of Iba1-positive cells was not affected in both sexes whereas less GFAP-positive cells were counted in the arcuate nucleus of male HFD + S mice, an effect that was completely reversed by Gln supplementation (Fig. 10E, F, G, p*-*values detailed in legends). However, the morphometric parameters of astrocytes were not affected by both chronic restraint stress and Gln in male obese mice (Supplemental Fig. S3). We although observed only a trend towards a decrease in the number of GFAP-positive cells (*p* = 0.08) and no effect of Gln in female HFD + S mice (Fig. 10F), further analysis revealed that the morphometric parameters of astrocytes (No. segment branch/terminal pts, Filament length sum, and Sholl intersections) were significantly reduced in female HFD + S mice compared to HFD mice, without additive effects of Gln supplementation (Fig. 11). Regarding microglial cells, we observed no differences in morphometric parameters except for their cell volume which tended to be higher with Gln supplementation in male obese mice under stress conditions (*p* = 0.0575, Supplemental Fig. S4). By contrast, there was a significant decrease in two parameters, i.e. Filament full branch depth in female HFD + S and the Sholl intersection number in female HFD + S + Gln mice (Supplemental Fig. S4).

## Discussion

Obesity is a major public health issue associated with several comorbidities. Recently, low levels of Gln, a non-essential amino acid, have been reported in plasma and adipose tissue during obesity [33]. Interestingly, experimental and some clinical data have shown that Gln administration can affect peripheral alterations (i.e. gut microbiota, gut barrier function, adipose tissue response, hepatic lipid accumulation) during obesity [1, 33, 49], Lefebvre et al., [24] bioRxiv preprint version). However, to our knowledge, no study has investigated the effects of Gln supplementation on the hypothalamic response to HFD although the hypothalamus plays a key role in the regulation of energy homeostasis and glycaemic response. Furthermore, previous data have reported a neuroinflammatory response in the hypothalamus during obesity [6, 17, 45]. Thus, in the present study, we report for the first time that Gln supplementation is able to affect the hypothalamic response during obesity in a sex-dependent manner, particularly under stress conditions.

Regarding the neuropeptides regulating food intake, both male and female obese mice exhibited an anorexigenic profile in response to HFD as previously described [13]. However, chronic restraint stress, which is associated with an increase in corticosterone in both sexes, induced changes in *Npy*, *Pomc* and *Mc4r* mRNA expression only in obese male mice corresponding to the changes in body weight. Multiple studies have demonstrated that stress strongly influences NPY expression in the brain and that its magnitude depends on the stress protocol and the delay between the stress stimulus and tissue collection. For instance, in Rat arcuate nucleus, *Npy* gene expression is increased after acute or chronic episodes of restraint stress [36]. However, only male animals were used in these studies [9, 20, 29, 44]. More recently, both male and female mice showed a body weight loss in response to a chronic social defeat stress, regardless of whether they were fed a SD or HFD [41]. In this latter study, *Npy* mRNA expression was increased only in female stressed mice on a standard chow diet, but not in males. However, the *Npy* mRNA expression in stressed mice on high-fat diet was not reported. In our study, in HFD mice, only male mice showed an increase in *Npy* mRNA and lost body weight. This discrepancy could be explained by the duration of HFD exposure, 14 weeks vs. 28 days. Interestingly, we observed a dissociation between *Npy* and *Agrp* expression in response to stress as previously described [20, 41]. NPY release in the hypothalamic paraventricular nucleus and basolateral amygdala has been associated with increased resilience to stress in male rats [30, 40], suggesting that the increase in *Npy* mRNA in stressed male mice could reflect a sustained compensatory mechanism to protect and recover from the stress. The melanocortin system, mainly through the MC4R has been demonstrated to play an important role in the regulation of energy homeostasis and the HPA axis activity [31]. In our present study, *Pomc* mRNA was affected by HFD only in female mice whereas chronic restraint stress reduced the level of *Pomc* mRNA only in male obese mice that was associated with an increase of *Mc4r* mRNA expression. Accordingly, in male rats exposed to chronic restraint stress for 12 days, an increase in *Mc4r* mRNA expression was previously reported in the arcuate nucleus [21] that was not reversed by the injection of glucocorticoid receptor antagonist. Finally, BDNF also contributes to modulate synaptic efficacy and to have neuroprotective and morphological effects after brain insults. An immobilization stress challenge is associated with an up-regulation in *Bdnf* mRNA expression in the hippocampus in adult male rats [27]. Although these finding were in hippocampus, BDNF is abundantly expressed in the brain including the hypothalamus. In our study, neither HFD nor chronic restraint stress in HFD mice modified *Bdnf* mRNA hypothalamic expression. Taken together, our results suggest that male obese mice were more vulnerable to our CRS model since both *Npy*, *Pomc* and *Mc4r* expression levels were only significantly affected in males compared to females, suggesting a possible habituation of the repeated stress exposure in females, even if plasma corticosterone was increased in both sexes.

Regarding the hypothalamic inflammatory response to HFD, our study also revealed a sex-specific effect of CRS in HFD mice. Indeed, in contrast to the neuropeptides regulating food intake, which were only affected in male mice, CRS mainly induced changes in hypothalamic inflammatory markers in female mice. Stress was associated with a decrease in *Il1β*, *Tnfα* and *Cd11b* mRNA levels in female obese mice whereas only *Il1β* was affected in males. Neither male nor female obese mice exhibited changes in microglial cell morphology in response to stress. However, only female obese mice showed altered astrocyte morphological parameters, even if the number of GFAP + cells was reduced in male HFD stressed mice. A study evaluating sex dependent effect of HFD and stress in the ventromedial hypothalamus by single-nuclei RNA sequencing analysis revealed increased microglial activation in female mice on HFD, whereas male mice on HFD showed astrocytic activation under repeated stress [38]. These effects were not observed in our model. The duration of the repeated stress, 2 h/day for 4 consecutive days compared to a weekly reminder shock for 14 weeks, could explain the discrepancy. The evaluation of the effects of a combination of stress and obesity in both sexes remains poorly documented and further research is needed.

In the present study, we also evaluated the impact of oral Gln supplementation on the hypothalamic response to HFD and HFD + S. Interestingly, Gln supplementation blunted the alterations of *Npy*, *Agrp* and *Crh* mRNA expression in unstressed male mice but not in female mice. However, Gln supply did not affect the mRNA expression of these neuropeptides under stress conditions, except for BDNF in males. In female mice, neither CRS nor Gln administration altered these neuropeptides mRNA expressions. NPY-induced food intake was reduced in chicks treated with the dipeptide Glycyl-Glutamine, considered as an endogenous antagonist of β-endorphin in several systems. This effect seemed to be mediated by the melanocortin system, as hypothalamic *Pomc* mRNA was decreased [39]. In our study, Gln supplementation also affected hypothalamic inflammatory response. Nevertheless, it seems that Gln supplementation increased *Il6* mRNA expression under HFD conditions and in stress conditions but only in female mice. IL6 is a rather unique cytokine that exerts pleiotropic actions in different organs and systems. It has been reported that IL6 actions in the hypothalamus protect against obesity and that IL6 is involved in the regulation of neurogenesis [7]. In female HFD stressed mice, Gln supplementation was also associated with a partial restoration of *Il1β* and *Tnfα* mRNA and an increase in *Iba1* mRNA levels. These effects were not observed in males. Accordingly, the evaluation of glial morphological parameters showed that Gln supplementation also induced a glial cell remodelling in a sex-dependent manner. Indeed, female mice supplemented with Gln had a reactive morphotype for microglial cells whereas a more complex astrocytic morphotype was revealed in male mice supplemented with Gln. These results support the previously highlighted sexual dimorphism of glial cells [10]. For instance, the response of astrocytes to saturated fatty acids, at least in vitro, is reported to differ between males and females suggesting that females transport and/or accumulate less palmitic acid in the brain under HFD conditions [8]. In the brain, astrocytes produce glutamine from excess glutamate and ammonia in order to protect neurons from excitotoxicity [43]. Microglial cells are able to use Gln as a metabolic fuel [19]. However, mice with an overexpression of the glutamine transporter SLC38A1 constitutively produced and excreted glutamate into the interstitial space, resulting in neurotoxicity [18]. Overall, our results suggest that Gln supplementation could play a role in the glial plasticity in the context of obesity and under stress conditions in a sex-specific fashion. Since Gln was administered at week 12 in our study, it should be also interesting to know whether Gln acts as a therapeutic agent or as a stress-buffering agent. This point needs furthers investigations. Nevertheless, the significance of this morphological remodelling remains to be further deciphered to know whether these changes translate into proinflammatory and/or neuroprotective mechanisms.

Some limitations of our study deserve discussions. Firstly, we were unable to evaluate precisely the dose of Gln received by each mouse, since mice were co-housed (*n* = 4/cage) for ethical reasons. Similarly, even if we did not observe significant differences in the food consumption by cage between Gln-treated and untreated mice (data not shown), we were not able to provide food intake for each mouse. Secondly, the immunostaining experiments are limited to a part of our animal cohorts and to a specific hypothalamic area, the arcuate nucleus. It would be interesting to explore the sex-specific impact of Gln and/stress on other hypothalamic areas such as the paraventricular nucleus, the ventromedial hypothalamic region and the lateral hypothalamic area that would allow to correlate the data of immunostaining to the data of RT-qPCR performed on the whole hypothalamus. Our study provides evidence of sex-specific glial responses to Gln and/stress. Finally, to better understand the underlying mechanisms, it could be of interest to evaluate the role of sexual hormones or of the estrous cycle. Further investigations in ovariectomized female mice or in castrated male mice should be done, as previously performed to evaluate the sex-specific response to high-fat diet on peripheral glucose tolerance (Gao et al. 2021).

In conclusion, our data highlight (i) that CRS exerts sex-specific effects on the hypothalamic response during DIO in mice and (ii) that Gln supplementation may affect neuropeptides regulating food intake and glial response in a sex-dependent manner. In particular, further studies are warranted to investigate the role of Gln-induced microglial reactivity in female obese mice.

## Supplementary Information

Below is the link to the electronic supplementary material.

Summary of statistical tests (XLSX 188 KB)

Schematic timeline of experimental protocols (JPG740 KB)

**Effect of Gln supplementation on hypothalamic glial markers expression in mice. **Graphs show the quantification of immunopositive cells for GFAP within the ARC from male and female mice fed with SD or HFD for 14 weeks which received or not Gln supplementation (experiment 1, N=2 images/animal with n=4 mice/group). Data were compared with nested *t* tests (**p<0.01) and are presented as mean ± standard error of the mean (SEM). Representative images show staining by immunofluorescence of GFAP+ cells manually and bilaterally counted within the ARC using Image J software in female mice (20 µm, -1.22 to 2.54 mm relative to Bregma). Scale bar : 20 µm, 3 V : third ventricle (TIF 1.64 MB)

Supplementary figure 12 (PNG 1.21 MB)

**Effect of stress and Gln supplementation on the morphological parameters of astrocytes in male mice.** Graphs show the values of each cell individually analyzed for Filament No. segment branch points/terminal points, Filament full branch depth/level, and Filament length sum (µm) in male mice fed with HFD for 14 weeks subjected or not to the stress which received or not Gln supplementation (N=2-3 cells/hemisphere from n=6 mice/group). Data were compared with nested *t*tests (*p<0.05) and are presented as mean ± standard error of the mean (SEM). Curves show the mean distribution of the number of Sholl intersections as a function of the distance from the astrocyte soma for male mice at week 14 (N=2-3 cells/hemisphere from 6 mice/group). Values were compared with 2-way ANOVA (group x distance) followed by Tukey’s multiple comparison tests (*p<0.05) (TIF 561 KB)

Supplementary figure 13 (PNG 283 KB)

**Effect of stress and Gln supplementation on the morphological parameters of microglial cells in mice.** Graphs show the values of each cell individually analyzed for Cell Volume (µm^3^), Filament full branch depth/level, Filament No. segment branch points/terminal points and Filament length sum (µm) in male and female mice fed with HFD for 14 weeks subjected or not to the stress and which received or not Gln supplementation (N=3-4 cells/hemisphere from n=6 mice/group). Data were compared with nested *t*tests (*p<0.05) and are presented as mean ± standard error of the mean (SEM). Curves show the mean distribution of the number of Sholl intersections as a function of the distance from the microglial cell soma for male and female mice at week 14 (N=3-4 cells/hemisphere from 6 mice/group). Values were compared with 2-way ANOVA (group x distance) followed by Tukey’s multiple comparisons test (*p<0.05). At 15 µm of distance from the soma, p=0.0568 HFD+S *vs*HFD+S+Gln; at 20 µm, p=0.0587 HFD+S *vs* HFD+S+Gln for female mice (TIF 1.15 MB)

Supplementary figure 14 (PNG 643 KB)

## Data Availability

No datasets were generated or analysed during the current study.

## Abbreviations

AgRP: Agouti-related protein
ARC: Arcuate nucleus
BDNF: Brain-derived neurotrophic factor
CRH: Corticotropin-releasing hormone
CRS: Chronic-restraint stress
DIO: Diet-induced obesity
GFAP: Glial fibrillary acidic protein
Gln: Glutamine
HFD: High-fat diet
HPA: Hypothalamic-pituitary-adrenal
Iba1: Ionized calcium-binding adapter molecule 1
IBS: Irritable bowel syndrome
IL: Interleukin
iNOS: Inducible form of nitric oxide synthase
MC4R: Melanocortin-4 receptor
NPY: Neuropeptide Y
PBS: Phosphate-buffered saline
PFA: Paraformaldehyde
POMC: Proopiomelanocortin
P2RY12: Purinergic receptor P2Y12
RT-qPCR: Reverse transcriptase quantitative polymerase chain reaction
SD: Standard diet
TNF: Tumor necrosis factor
