# Supplementary figures and images for: Comparative Analysis of Hypothalamic Responses to Stress and Glutamine Supplementation in Diet-Induced Obese Mice: A Study of Sex Differences
Source: Inflammation. 2026 Jan 10;49(1):37. doi: 10.1007/s10753-025-02428-9 (PMC12858480; doi:10.1007/s10753-025-02428-9)

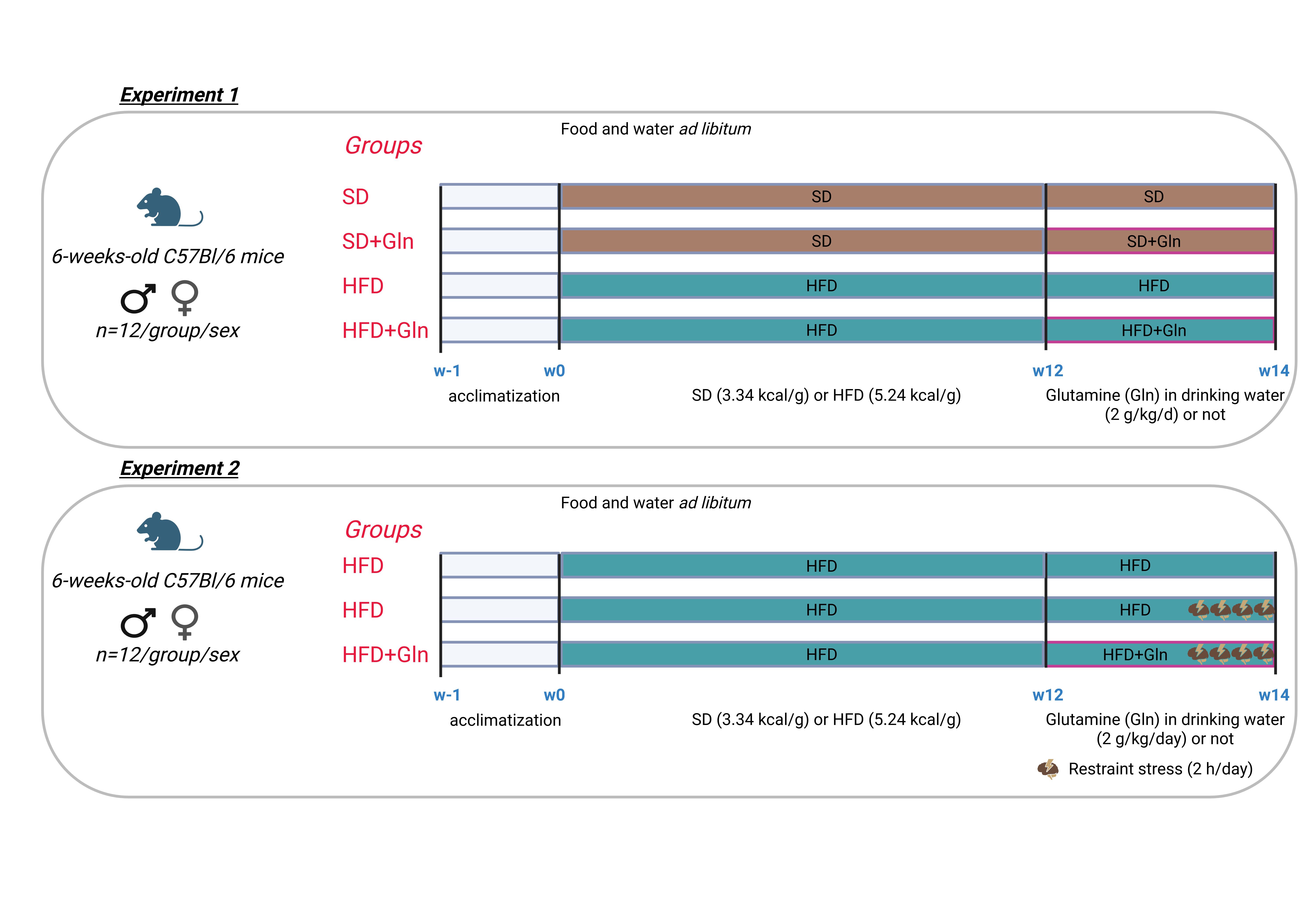

Supplement: Supplementary file 2 — Schematic timeline of experimental protocols (JPG740 KB) [file 10753_2025_2428_MOESM2_ESM.jpg]

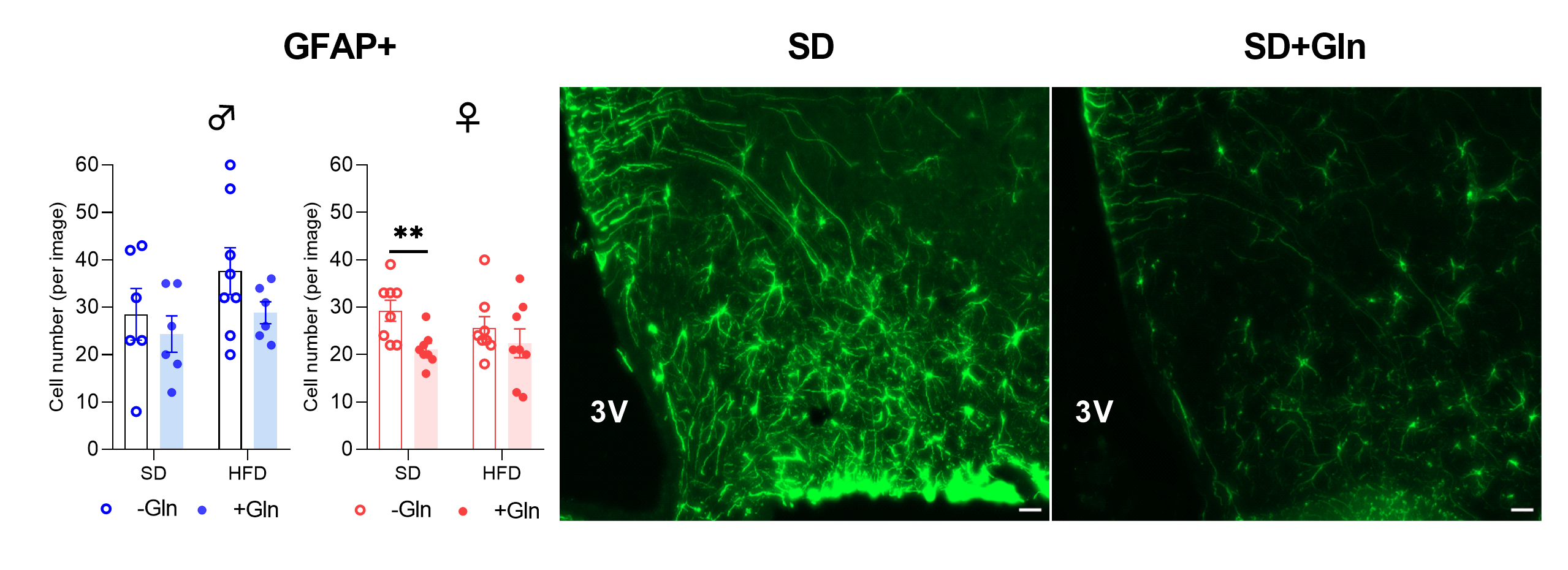

Supplement: Supplementary file 3 — Effect of Gln supplementation on hypothalamic glial markers expression in mice. Graphs show the quantification of immunopositive cells for GFAP within the ARC from male and female mice fed with SD or HFD for 14 weeks which received or not Gln supplementation (experiment 1, N=2 images/animal with n=4 mice/group). Data were compared with nested t tests (**p<0.01) and are presented as mean ± standard error of the mean (SEM). Representative images show staining by immunofluorescence of GFAP+ cells manually and bilaterally counted within the ARC using Image J software in female mice (20 µm, -1.22 to 2.54 mm relative to Bregma). Scale bar : 20 µm, 3 V : third ventricle (TIF 1.64 MB) [file 10753_2025_2428_MOESM3_ESM.tif]

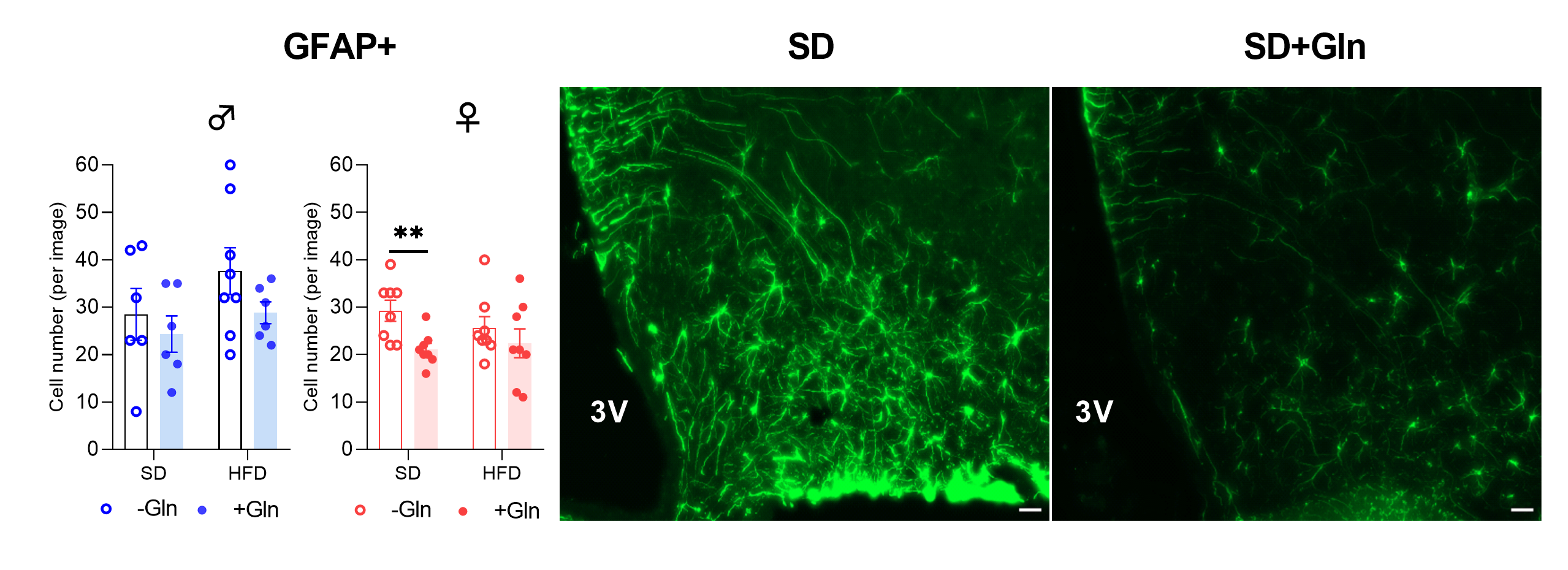

Supplement: Supplementary file 4 — (PNG 1.21 MB) [file 10753_2025_2428_Fig12_ESM.png]

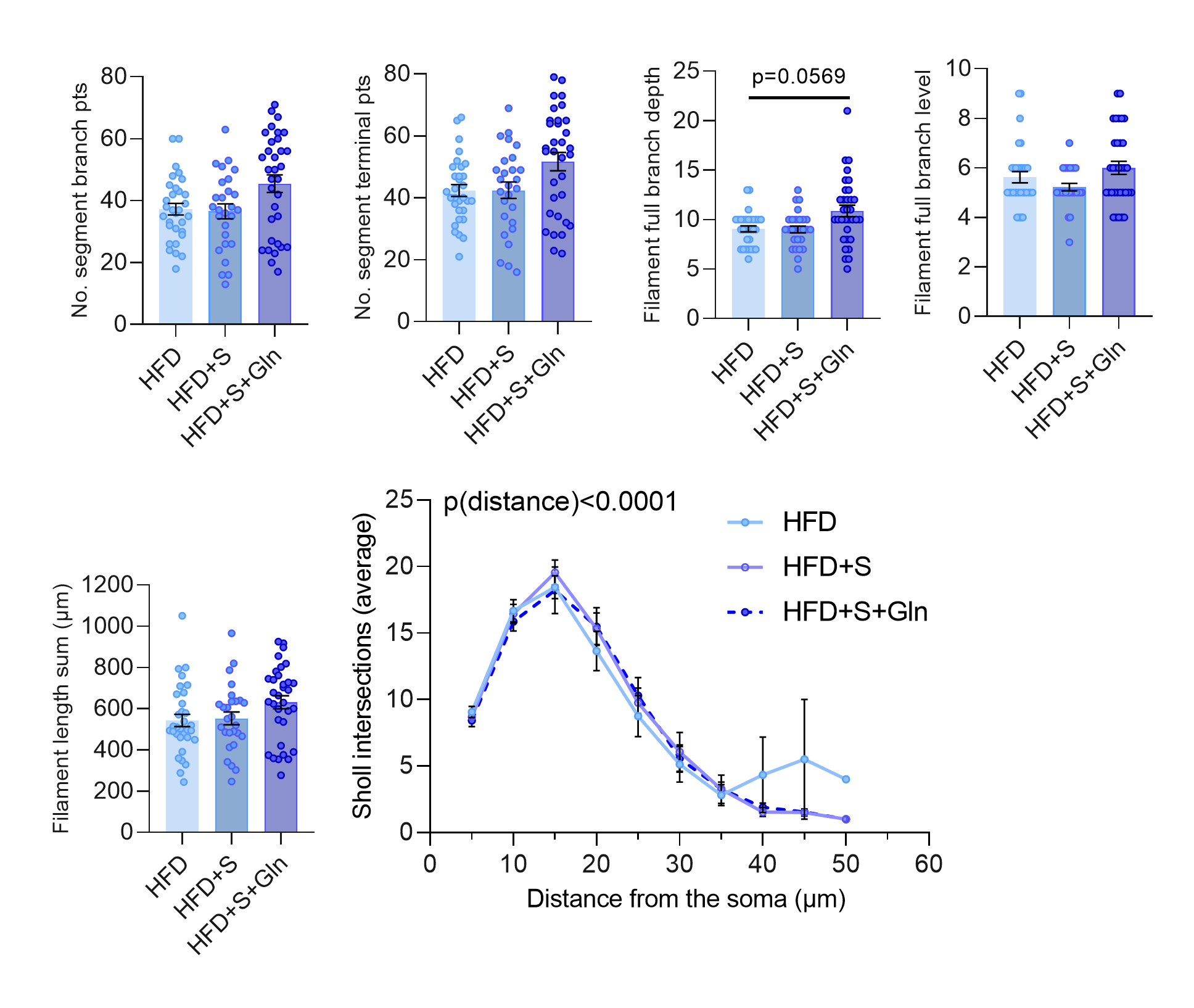

Supplement: Supplementary file 5 — Effect of stress and Gln supplementation on the morphological parameters of astrocytes in male mice. Graphs show the values of each cell individually analyzed for Filament No. segment branch points/terminal points, Filament full branch depth/level, and Filament length sum (µm) in male mice fed with HFD for 14 weeks subjected or not to the stress which received or not Gln supplementation (N=2-3 cells/hemisphere from n=6 mice/group). Data were compared with nested ttests (*p<0.05) and are presented as mean ± standard error of the mean (SEM). Curves show the mean distribution of the number of Sholl intersections as a function of the distance from the astrocyte soma for male mice at week 14 (N=2-3 cells/hemisphere from 6 mice/group). Values were compared with 2-way ANOVA (group x distance) followed by Tukey’s multiple comparison tests (*p<0.05) (TIF 561 KB) [file 10753_2025_2428_MOESM4_ESM.tif]

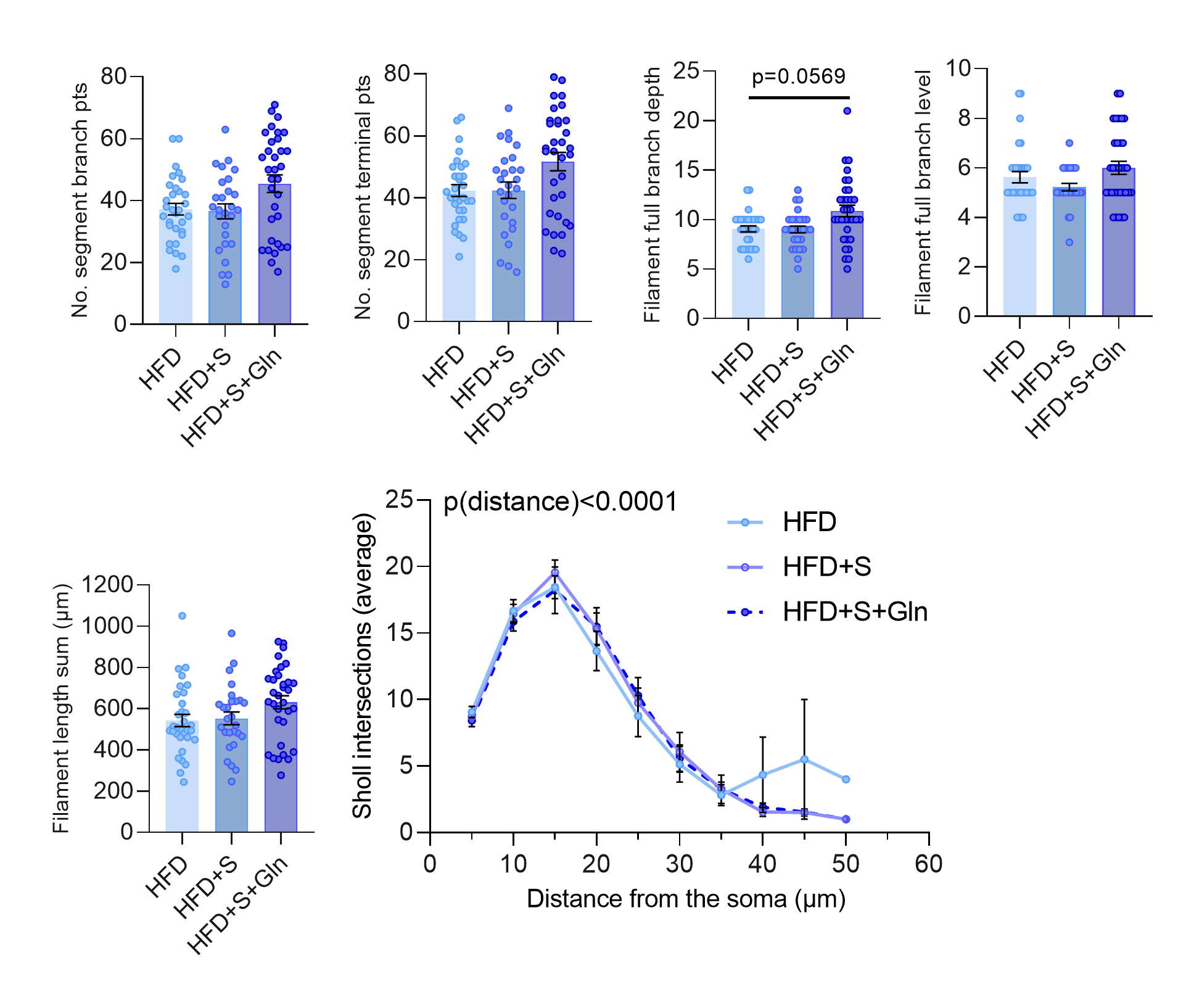

Supplement: Supplementary file 6 — (PNG 283 KB) [file 10753_2025_2428_Fig13_ESM.png]

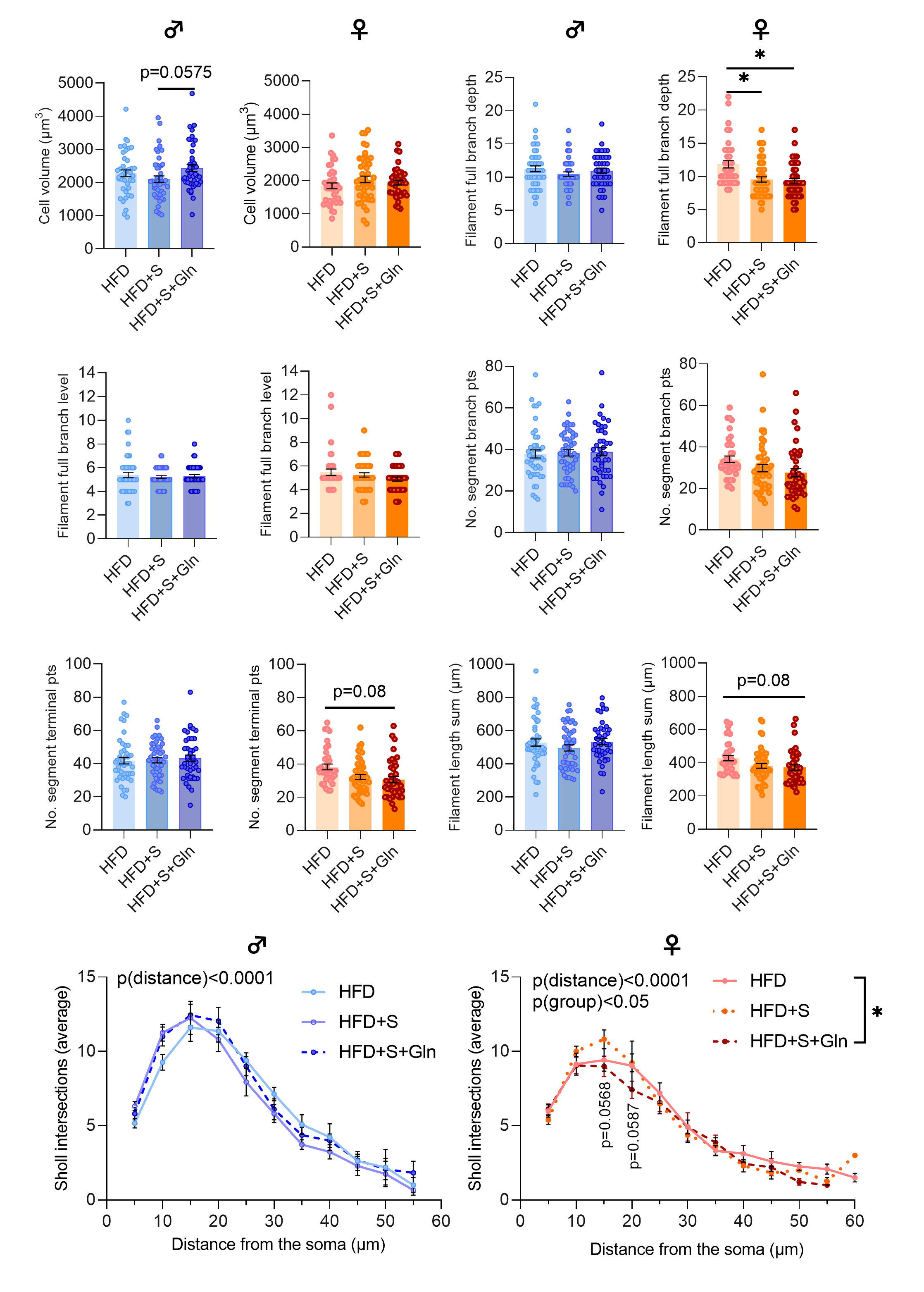

Supplement: Supplementary file 7 — Effect of stress and Gln supplementation on the morphological parameters of microglial cells in mice. Graphs show the values of each cell individually analyzed for Cell Volume (µm3), Filament full branch depth/level, Filament No. segment branch points/terminal points and Filament length sum (µm) in male and female mice fed with HFD for 14 weeks subjected or not to the stress and which received or not Gln supplementation (N=3-4 cells/hemisphere from n=6 mice/group). Data were compared with nested ttests (*p<0.05) and are presented as mean ± standard error of the mean (SEM). Curves show the mean distribution of the number of Sholl intersections as a function of the distance from the microglial cell soma for male and female mice at week 14 (N=3-4 cells/hemisphere from 6 mice/group). Values were compared with 2-way ANOVA (group x distance) followed by Tukey’s multiple comparisons test (*p<0.05). At 15 µm of distance from the soma, p=0.0568 HFD+S vsHFD+S+Gln; at 20 µm, p=0.0587 HFD+S vs HFD+S+Gln for female mice (TIF 1.15 MB) [file 10753_2025_2428_MOESM5_ESM.tif]

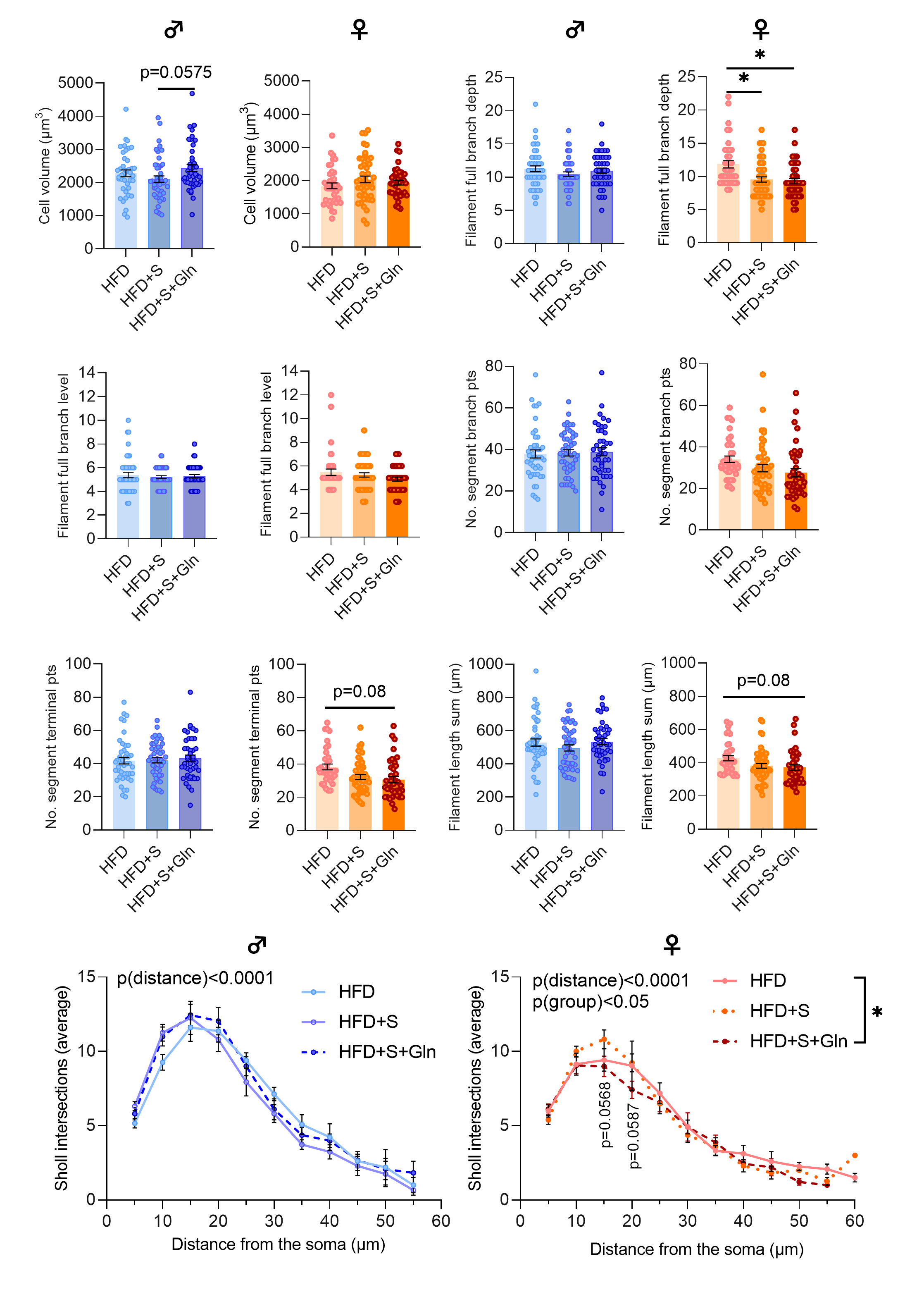

Supplement: Supplementary file 8 — (PNG 643 KB) [file 10753_2025_2428_Fig14_ESM.png]
